# Integrated multi-omics analysis reveals a gut microbiota–tryptophan metabolism axis contributes to sex differences in a β-aminopropionitrile-induced aortic dissection mouse model

**DOI:** 10.1186/s13293-026-00925-6

**Published:** 2026-05-20

**Authors:** Shuai Cheng, Xinyu Hao, Shirui Liu, Linfeng Zhang, Shuai Zhang, Yutian Chen, Lei Wang, Shijie Xin, Zhen Li, Zhaohui Hua, Hui Cao

**Affiliations:** 1 Department of Endovascular Surgery, The First Affiliated Hospital of Zhengzhou University, No.1, East Jian She Road, Zhengzhou, 450052 Henan Province China; 2 Department of Vascular and Thyroid Surgery, The First Hospital of China Medical University, Shenyang, Liaoning Province China

**Keywords:** Aortic dissection, Sex differences, Multi-omics, Tryptophan metabolism, Indolepyruvate

## Abstract

**Background:**

Sex differences in aortic dissection (AD) have been consistently reported in epidemiological studies and experimental mouse models, with males showing markedly higher susceptibility. However, the molecular basis underlying these sex-specific differences remains insufficiently understood.

**Methods:**

Three-week-old male and female C57BL/6J mice were administered 0.4% β-aminopropionitrile (BAPN) in drinking water for 28 d to induce AD. After the induction period, fecal samples, serum, and aortic tissues were collected from all surviving animals. Integrated analyses included strand-specific transcriptomic sequencing of aortic tissues, untargeted serum metabolomics, and full-length 16 S rRNA sequencing of fecal samples to characterize sex-related differences across transcriptomic, metabolic, and microbiome layers. Inter-omics correlations were further assessed using bioinformatic approaches. Furthermore, in vivo experiments were conducted to validate the impact of key metabolites on the progression of AD.

**Results:**

Female mice exhibited significantly lower susceptibility to BAPN-induced AD, including reduced rates of aortic rupture, lower incidence of AD or aneurysm (AAD), and attenuated aortic dilation. Transcriptomic analysis revealed that female non-dissected mice (FeNonAD) displayed diminished induction of inflammation-related genes and lower predicted immune cell infiltration. Metabolomic profiling revealed significant elevations of tryptophan–indole pathway metabolites—such as indolepyruvate, indole-3-acetic acid, and indolepropionic acid—in both FeNonAD and AAD groups. Microbiome analysis further revealed a higher relative abundance of tryptophan-metabolizing taxa, particularly key *Clostridium* species, in the intestinal tract of FeNonAD mice, accompanied by significant upregulation of key functional genes (*tyrB* and *aspC*) associated with indolepyruvate synthesis. Weighted gene co-expression network analysis (WGCNA)-based integration identified strong negative correlations between indolepyruvate and indole-3-acetic acid sodium salt levels and aortic gene modules linked to immune-inflammatory activation. Further in vivo experiments demonstrated that treatment with indolepyruvate delayed AD progression in male mice.

**Conclusion:**

This study highlights a central “gut microbiota–tryptophan metabolism–aortic inflammation” axis that contributes to sexual dimorphism in BAPN-induced AD. These findings provide new molecular insights into sex-specific disease mechanisms and offer a conceptual basis for developing sex-tailored diagnostic and therapeutic strategies.

**Supplementary Information:**

The online version contains supplementary material available at 10.1186/s13293-026-00925-6.

## Abstract (plain language summary)

Aortic dissection is a dangerous condition, in which the wall of the main artery leaving the heart suddenly tears. It leads to internal bleeding and can be fatal. Doctors have long known that men develop this condition more often than women, but the reasons behind this difference have remained unclear.

We used a mouse model to investigate why females appear to be better protected against aortic dissection. We examined several layers of biology simultaneously, including gene activity in the aorta, circulating chemicals, and bacteria in the gut. We aimed to understand how the body responds before and during disease.

Female mice showed fewer signs of inflammation in the aorta and were less prone to vessel rupture. Their blood also contained higher levels of certain molecules made from the amino acid tryptophan. These molecules, called indole metabolites, are known to modulate immune responses and support healthy cell function. We discovered that female mice produced more of these protective molecules because their gut bacteria were better at converting tryptophan into indole compounds. In further experiments, supplementing male mice with indolepyruvate, an indole metabolite, reduced the incidence of aortic dissection.

We found that these indole metabolites were associated with reduced activation of inflammation-related genes in the aorta. This suggests that communication between the gut and the blood vessel wall may help protect females from disease.

Our findings highlight a gut–body pathway that may explain why females are less vulnerable to aortic dissection and point to new possibilities for prevention or treatment.

**Supplementary Information:**

The online version contains supplementary material available at 10.1186/s13293-026-00925-6.

## Highlights

Female mice were more resistant to aortic dissection than male mice, with fewer ruptures and less aortic injury.

Female mice showed distinct shifts in blood levels of tryptophan-related chemicals, producing higher levels of protective indole molecules even before visible disease developed. Treatment with indolepyruvate suppressed AD progression in male mice.

Gut bacteria that help convert tryptophan into protective indole compounds were more active in female mice, suggesting a strong link between gut microbes and vascular health.

Combining all data showed that these indole molecules were connected to lower inflammation and better cell maintenance in the aorta, revealing a gut–tryptophan pathway that may help explain why females are less vulnerable to aortic dissection.

**Supplementary Information:**

The online version contains supplementary material available at 10.1186/s13293-026-00925-6.

## Background

Aortic dissection (AD) is a catastrophic cardiovascular emergency characterized by sudden onset and extremely high mortality. Its pathological hallmark is the entry of blood through an intimal tear into the medial layer of the aortic wall, forming a false lumen. This false lumen can propagate longitudinally along the aorta, potentially involving major branch vessels and leading to life-threatening complications such as organ ischemia, cardiac tamponade, or aortic rupture. The acute onset of AD is associated with various factors, including conditions that weaken the vascular wall structure (e.g., connective tissue disorders, atherosclerosis, infectious or immune-mediated aortitis) and triggers that cause sudden, severe fluctuations in blood pressure (e.g., uncontrolled hypertension, emotional stress, or strenuous physical activity) [1, 2]. Multiple key molecular events, including endothelial dysfunction, smooth muscle cell phenotypic switching, programmed cell death, and immune cell infiltration, underlie the initiation and progression of AD. However, the specific molecular regulatory networks governing these events remain insufficiently clarified [3–6].

Because of physiological, anatomical, and hormonal differences, susceptibility to specific diseases varies between sexes. Research indicates that sex substantially influences the clinical manifestations and pathogenesis of cardiovascular diseases [7]. For instance, in atherosclerosis, coronary artery disease, myocardial infarction, and heart failure, men and women differ in incidence, pathological presentation, and therapeutic responses. Generally, women have a lower risk of coronary artery disease, ischemic myocardial injury, and abdominal aortic aneurysm, yet a higher risk of spontaneous coronary artery dissection and Takotsubo cardiomyopathy (“broken heart syndrome”) [8, 9]. Epidemiological data indicate that AD incidence is significantly higher in men than in women [10]. Women with AD are typically older at the time of diagnosis and present with more comorbidities [11]. Although these findings remain inconsistent, some studies suggest that women may experience poorer outcomes after open surgical repair [12]. Nevertheless, the underlying cellular and molecular mechanisms of sex-related differences in AD remain poorly understood.

Mouse models of AD, widely used to investigate disease mechanisms, also exhibit intrinsic sexual dimorphism. β-aminopropionitrile (BAPN) is the most commonly used chemical agent to experimentally induce AD. By disrupting collagen and elastin cross-linking, BAPN compromises arterial wall integrity and reproduces key pathological characteristics of human AD [13]. It may be administered alone or combined with other factors, such as angiotensin II (AngII) or a high-fat diet, to establish experimental models [14]. In experimental models combining BAPN and AngII, female mice have demonstrated significantly reduced susceptibility to AD [15]. More recent findings show that in BAPN-only models, female mice consistently exhibit lower AD incidence and reduced rupture rates throughout a 4-week induction period [16, 17]. Therefore, elucidating the mechanisms underlying these sex-based differences is essential for advancing understanding of AD biology and developing precise diagnostic and individualized therapeutic strategies.

Advances in high-throughput sequencing, mass spectrometry, and bioinformatics have enabled omics technologies to provide unprecedented depth and breadth for disease research. Genomics, transcriptomics, proteomics, and metabolomics enable systematic characterization of molecular alterations during disease progression at the DNA, RNA, protein, and metabolite levels, respectively [18]. In AD studies, metabolomics analyses have identified distinctive metabolic disturbances in patients with acute aortic dissection (AAD), including alterations in circulating succinate, lipid species, and taurine, changes implicated in disease progression [19–22]. Microbiome research has also shown that patients with AD exhibit gut microbial dysbiosis, including increases in *Prevotella* and *Klebsiella* and decreases in beneficial *Bacteroides*, which may contribute to disease development by modulating host metabolism and impairing intestinal barrier integrity [23–25]. In recent years, integrative multi-omics approaches have uncovered novel mechanisms linking ionic homeostasis imbalance with immune activation in AD development [26, 27]. Because multi-omics strategies generate comprehensive and mutually corroborative data, they have become an important direction in contemporary AD research.

This study employed full-length 16 S rRNA sequencing, strand-specific transcriptomic sequencing, and untargeted metabolomics to characterize the multi-omics profiles of BAPN-induced AD mouse models of both sexes. Through an integrated analysis, we aimed to delineate the sex-dependent molecular landscape of AD progression and to explore potential mechanisms underlying the protective effect observed in the female sex against AD.

## Methods

### Establishment of the BAPN-induced AD model and grouping

Three-week-old male and female C57BL/6J mice were obtained from GemPharmatech Co., Ltd. (Jiangsu, China). The sample size was determined based on previous literature reporting a 70–80% AD incidence and an approximate 40–50% mortality rate in BAPN-treated male mice, along with the requirement of 6–10 biological replicates per group for robust multi-omics analysis [14]. A formal power analysis was not performed due to the lack of reliable incidence data in females. To account for the high anticipated mortality, initial group sizes were set at *N* = 20 for BAPN-treated males and *N* = 19 for BAPN-treated females. This study was conducted and reported in accordance with the ARRIVE guidelines. All animals were maintained in the specific pathogen-free facility of the Experimental Animal Center at Zhengzhou University, under controlled environmental conditions (20–24 °C; stable humidity) and a 12-h light/dark cycle, with ad libitum access to food and water. After a 3-d acclimatization, mice were randomly allocated into four groups and ear-tagged as follows:

1. **MaCon (N = 10)**: Male mice receiving normal drinking water.
2. **MaBAPN (N = 20)**: Male mice receiving 0.4% BAPN in drinking water.
3. **FeCon (N = 10)**: Female mice receiving normal drinking water.
4. **FeBAPN (N = 19)**: Female mice receiving 0.4% BAPN in drinking water.

In a separate set of experiments which aimed to evaluate the effects of indolepyruvate (I3P), three-week-old male C57BL/6J mice were randomly assigned to three groups: ① Con (*N* = 6), receiving normal drinking water; ② AD + Veh (*N* = 16), receiving 0.4% BAPN in drinking water and intraperitoneal (i.p.) injections of vehicle (10% DMSO + 40% PEG300 + 5% Tween-80 + 45% Saline); ③ AD+I3P (*N* = 16), receiving 0.4% BAPN in drinking water and i.p. injections of I3P (20 mg/kg, MCE HY-W028393, every other day).

Throughout the 4-week induction period, animals were monitored three times daily. Additionally, individual body weights were recorded weekly for all groups, and weekly water consumption was recorded for the BAPN-treated groups. Any mouse that died from aortic rupture (Male Rupture/Female Rupture) underwent immediate necropsy, and the aortic tissues were collected and documented. At the end of the induction period, all surviving mice were euthanized and reclassified into the following groups based on the pathological outcomes:

1. **MaCon**: Male control group.
2. **MaNonAD**: Male BAPN-treated mice without microscopically detectable aortic lesions.
3. **MaAAD**: Male BAPN-treated mice that developed AD or aneurysm.
4. **FeCon**: Female control group.
5. **FeNonAD**: Female BAPN-treated mice without microscopically detectable aortic lesions.
6. **FeAAD**: Female BAPN-treated mice that developed aortic dissection or aneurysm.

### Sample collection

For the multi-omics characterization of the male and female cohorts, all surviving mice at the study endpoint (*N* = 48) were utilized across all multi-omics platforms (transcriptomics, metabolomics, and 16 S rRNA sequencing) without any sub-setting. All sample collection and subsequent experimental procedures were performed blinded to the experimental group allocation. To minimize potential cage effects on the gut microbiome, the animals were distributed across multiple cages within each group (4 cages per group for BAPN-treated males and females; two cages per group for male and female controls). Male and female mice were housed separately to prevent mating, but were maintained in the same SPF room and received the same batch of diet and water. Fecal samples for downstream analyses were collected from mice across all cages to avoid cage-specific bias.

### Fecal samples

All fecal samples were collected on the same day at the endpoint of the 4‑week induction, within a consistent morning time window and immediately before euthanasia. Fresh fecal pellets were collected from manually restrained mice as they naturally defecated. Approximately 3–4 pellets were obtained per mouse into sterile, nuclease-free 2 mL tubes without contact with bedding or other contaminants. Samples were snap-frozen in liquid nitrogen and stored at − 80 °C until DNA extraction. Samples from all cages and both sexes were collected in a randomized order by the same investigator. This standardized procedure was applied uniformly across all groups to ensure comparability.

### Serum samples

After deep isoflurane anesthesia (confirmed by unconsciousness and loss of reflexes), venous blood was obtained via retroorbital bleeding. Samples were subjected to double centrifugation (3,000 rpm, 10 min each, 4 °C), and the resulting serum was aliquoted, labeled, and rapidly frozen at − 80 °C until analysis.

### Aortic tissue

Mice were euthanized via anesthetic overdose, and death was confirmed by thoracotomy. The right auricle was incised, and systemic perfusion was performed via left ventricular puncture using 10 mL pre-cooled physiological saline. The thoracic aorta (ascending aorta, aortic arch, and proximal descending aorta) was rapidly dissected, photographed at 20× magnification, excised, snap-frozen in liquid nitrogen, and stored at − 80 °C. For the I3P treatment groups, mice were similarly euthanized and subjected to cardiac perfusion with 4% paraformaldehyde (PFA) for fixation before tissue harvest. Gross aortic images of both fixed and fresh samples were analyzed in ImageJ (v1.54p) to measure the maximum diameter of each segment. Tissues intended for multi-omics analysis were snap-frozen in liquid nitrogen without fixation. The PFA-perfused samples from I3P treatment groups were used exclusively for histological and morphometric assessments.

### Strand-specific transcriptomic sequencing and analysis

Total RNA from lesioned thoracic aortic segments was isolated using an RNeasy Micro Kit (QIAGEN, Germany) according to the manufacturer’s instructions. Strand-specific RNA-seq libraries were prepared from enriched mRNA and sequenced on the DNA NanoBall Sequencing platform. Subsequently, a comprehensive bioinformatics analysis was performed. Bray–Curtis distances between samples were calculated using the “vegan (v2.6-4)” package in R, followed by Principal Coordinates Analysis (PCoA) and PERMANOVA testing. Homogeneity of multivariate dispersions was additionally assessed using PERMDISP (betadisper function, vegan v2.6-4), with pairwise comparisons corrected by the Benjamini–Hochberg (BH) procedure. Differential gene expression analysis was performed on count data using the “DESeq2” package. Genes meeting the criteria of |LogFC| ≥ 1 and BH-adjusted *p* < 0.05 were defined as differentially expressed genes, and multi-group volcano plots were visualized with the “scRNAtoolVis (v0.1.0)” package. Pattern clustering analysis was conducted using the “Mfuzz (v2.62.0)” package, which utilizes a fuzzy c-means algorithm to group molecules with similar expression profiles. Functional enrichment analyses, including Gene Ontology (GO) and the Kyoto Encyclopedia of Genes and Genomes (KEGG) pathway analysis, were performed using the “clusterProfiler (v4.10.1)” R package with BH adjustment for multiple testing, and terms with an adjusted *p* < 0.05 were considered significantly enriched. Transcription factor enrichment analysis was conducted via the Metascape database (https://metascape.org/). Immune cell infiltration was estimated using the “ImmuCellAI-mouse (v0.1.0)” package.

### Untargeted metabolomics sequencing and analysis

Serum metabolites were extracted using a solvent containing internal standards (20 mg/L). Following incubation at − 20 °C, samples were centrifuged (12,000 × g, 15 min, 4 °C), and 500 µL of supernatant was vacuum-dried. Dried extracts were reconstituted in 160 µL acetonitrile/water (1:1, v/v), vortexed for 30 s, ultrasonicated on ice, and centrifuged again. A 120 µL aliquot of supernatant was transferred to autosampler vials for analysis. Quality control (QC) samples were prepared by pooling 10 µL aliquots from each processed sample.

Metabolic profiling was conducted using an ultra-performance liquid chromatography tandem mass spectrometry UPLC–MS/MS system (UPLC: Waters Acquity I-Class PLUS; MS: Waters Xevo G2-XS QTof). Raw data were acquired using MassLynx V4.2 (Waters). Data processing, including peak extraction and alignment, was performed using the Progenesis QI software (Waters). Metabolite identification was achieved by matching accurate mass, isotope distribution, and MS/MS fragmentation patterns against the METLIN database, the Human Metabolome Database (HMDB), the KEGG Compound database, the Lipid Maps Structure Database (LMSD), and an extensively curated in-house spectral library (BP ZJK, Biomarker Technologies, Beijing, China). According to the Metabolomics Standards Initiative (MSI) guidelines, the identified metabolites met the level 2 criteria. The resulting data matrices were log10-transformed and auto-scaled. Differential and enrichment analysis, including orthogonal partial least squares discriminant analysis [OPLS-DA] and KEGG pathway analysis, were conducted using MetaboAnalyst 6.0 (https://www.metaboanalyst.ca/). The significance of differences in individual metabolites was assessed using Student’s t-test, and Variable Importance in Projection (VIP) scores were derived from the OPLS-DA model. Differentially expressed metabolites (DEMs) were identified using thresholds of VIP > 1 and *p-*value < 0.05. Pathways identified through KEGG enrichment with *p*-values < 0.05 were considered significant. Beta diversity was assessed by calculating Bray–Curtis distances between samples using the “vegan (v2.6-4)” R package, followed by PCoA and PERMANOVA testing. The “scRNAtoolVis (v0.1.0)” R package was used to visualize multi-group differential analyses in volcano plots. To characterize the dynamic molecular trajectories during disease progression (Con, NonAD, and AAD), pattern clustering analysis of DEMs was conducted using the “Mfuzz (v2.62.0)” R package. Heatmap visualization of DEMs was generated using the “pheatmap (v1.0.12)” package.

### High-throughput 16 S ribosomal RNA gene sequencing

Genomic DNA was isolated from fecal samples using the TGuide S96 Magnetic Soil/Stool DNA Kit (Tiangen Biotech, Beijing, China) according to the manufacturer’s instructions. The hypervariable V1–V9 regions of the bacterial 16 S rRNA gene were amplified using universal primers 27 F (AGRGTTTGATYNTGGCTCAG) and 1492R (TASGGHTACCTTGTTASGACTT). Amplicons were quantified, pooled at equimolar concentrations, and sequenced on a PacBio Sequel II platform (Biomarker Technologies, Beijing). Bioinformatic analysis was performed using the BMK Cloud platform (http://www.biocloud.net/). Using USEARCH (v10.0.240_i86), quality-filtered sequences were grouped into OTUs based on a 97% sequence similarity threshold. Taxonomic assignment of OTUs was conducted in QIIME2 (v2020.6) using the naïve Bayes classifier with the SILVA database (Release 138.1) at a 70% confidence threshold. To ensure taxonomic accuracy at the species level, particularly for key differentially abundant OTUs, representative sequences were extracted and manually verified using the Basic Local Alignment Search Tool (BLASTn) against the NCBI nucleotide database. Taxonomic annotations were subsequently corrected based on the highest sequence identity. Alpha diversity (Chao1, PD_whole_tree, Shannon, Simpson) indices were calculated in QIIME2 (v2020.6) to evaluate within-sample species diversity. Beta diversity was assessed using Bray–Curtis, Weighted UniFrac, and Unweighted UniFrac distances, followed by Principal Coordinates Analysis (PCoA), PERMANOVA testing, and PERMDISP analysis with BH-corrected pairwise comparisons. Differential abundance analysis of taxa across groups was conducted using Linear Discriminant Analysis Effect Size (LEfSe v 1.1.1). LEfSe first applies the non-parametric Kruskal–Wallis sum-rank test to detect features with significant differential abundance among groups. To strictly control for false discoveries, the raw *p*-values were subsequently adjusted for multiple comparisons using the BH method (adjusted *p* < 0.05 was considered statistically significant). This was followed by pairwise Wilcoxon tests between subclasses (α = 0.05). Finally, Linear Discriminant Analysis (LDA) was used to estimate the effect size of each differentially abundant feature, and features with an LDA score > 2.0 were considered enriched.

The metabolic functional potential of the microbiota was predicted based on OTUs using PICRUSt2 (v2.3.0). In addition, count-based differential abundance analysis was performed at the phylum, genus, and species levels, as well as for KEGG Level 3 pathways and KEGG orthology (KO) entries, using DESeq2 (v1.42.1) and PICRUSt2. A significance threshold of *p* < 0.05 was applied. Differential analysis visualization was performed using the “scRNAtoolVis (v0.1.0)” R package, and correlation scatter plots were generated using the “GGally (v2.4.0)” package. To ensure the consistency and robustness of our findings, a parallel analysis was conducted using the DADA2 (1.20.0) pipeline. This independent verification included re-evaluating beta diversity (Bray–Curtis distance with PERMANOVA), LEfSe biomarkers, and PICRUSt2 functional profiles based on exact Amplicon Sequence Variants (ASVs).

### Multi-omics integrated analysis

Weighted Gene Co-expression Network Analysis (WGCNA) was performed using the WGCNA (v1.72-1) R package to investigate aortic gene expression patterns associated with serum indole metabolites. The soft-thresholding power (β) was determined using the pickSoftThreshold function, with a signed network specified. A β value yielding a scale-free topology fit index of R² > 0.9 was selected as the optimal soft threshold. The minimum module size was set to 100, and modules with similar expression profiles were merged using a dissimilarity threshold of 0.25, resulting in the final gene modules. Spearman’s correlation analysis was conducted between module eigengenes and the expression levels of differential serum indole metabolites. Functional enrichment analysis for genes within significant modules was performed using the “clusterProfiler (v4.10.1)” R package, and enriched terms with an adjusted *p*-value < 0.05 were considered statistically significant and retained for visualization. Categorical variables were compared using Fisher’s exact test.

### Histological staining and analysis

Aortic tissues were embedded in paraffin and cut into 3-µm-thick sections. Hematoxylin and Eosin (H&E) and Elastin Van Gieson (EVG) staining were performed to evaluate the histological morphology of the aorta. The degradation of elastic fibers was assessed using an EVG staining score as previously described [28].

### Statistical analysis

Statistical analyses were carried out using R (v4.3.2), with continuous variables summarized as mean ± standard deviation. Before inter-group comparisons, data distribution and variance homogeneity were examined using the Shapiro–Wilk and Levene’s tests, respectively. Accordingly, the Mann–Whitney U test was employed for non-normally distributed data. Comparisons involving normally distributed variables utilized either Student’s t-test (under equal variances) or Welch’s t-test (under unequal variances). Categorical variables were analyzed using Fisher’s exact test.

## Results

### Female mice exhibit reduced susceptibility to BAPN-induced AD

**Fig. 1:**
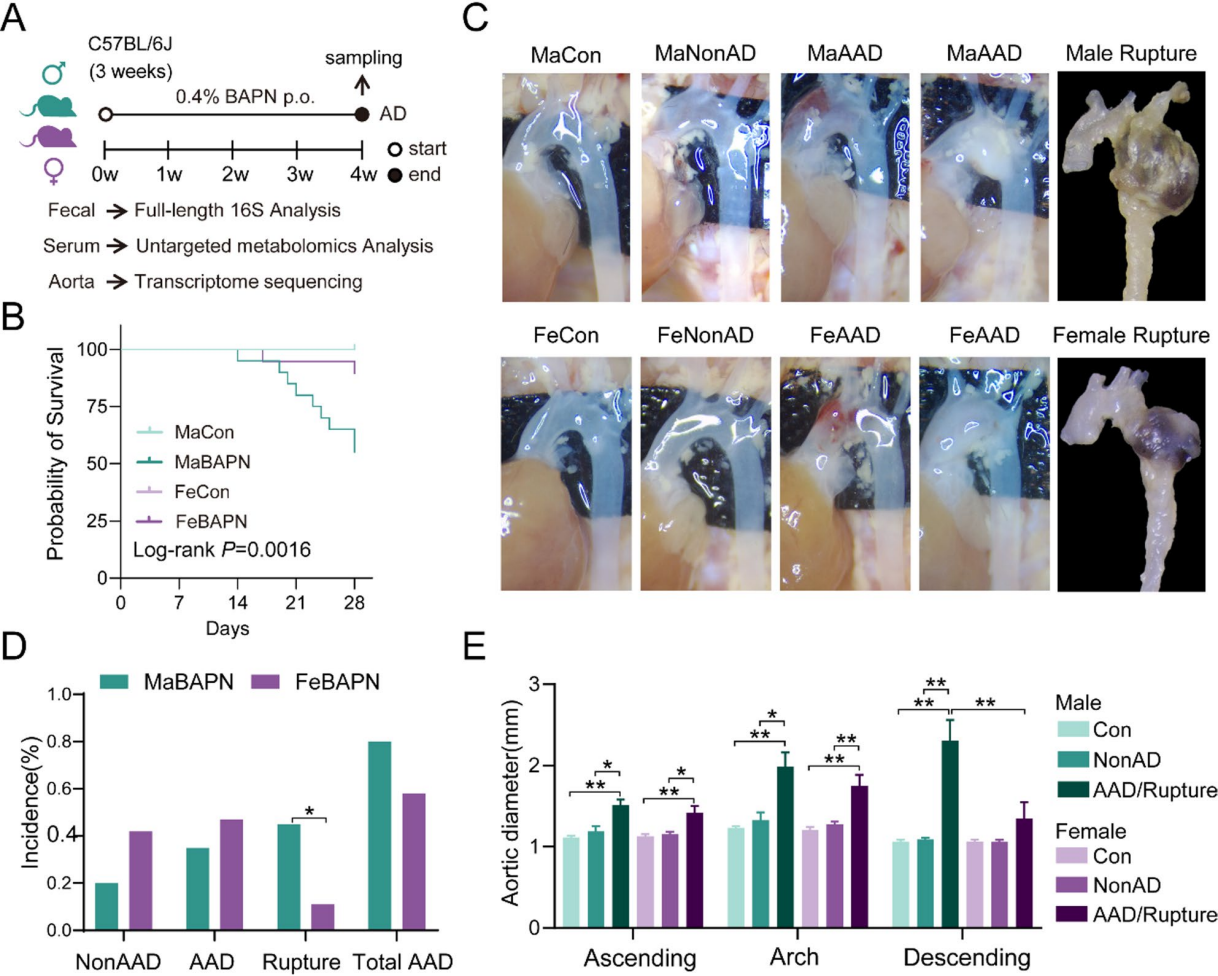
Establishment of the β-aminopropionitrile (BAPN)-induced aortic dissection (AD) model and comparison between sexes. (**A**) Experimental flowchart of the study. (**B**) Survival curves of different groups. (**C**) Representative aortic images for each group. (**D**) Statistical comparison of outcomes between male and female mice. (**E**) Comparison of aortic segment diameters between sexes. * *p* < 0.05, ** *p* < 0.01

The experimental workflow is shown in Fig. 1A. During the 4-week induction, both male and female mice maintained steady body weight gain (Figure S1A). Although female mice had slightly lower absolute water consumption, their normalized water intake was comparable to that of males (Figure S1B, C). This indicates that there was no significant difference in BAPN intake between males and females. Throughout the 4-week BAPN induction period, female mice exhibited significantly greater resistance to aortic rupture than did male mice, as reflected by the markedly improved survival rate in the FeBAPN group compared to that in the MaBAPN group (Fig. 1B). Pathological assessment at the end of the induction period revealed that the lesions were primarily localized to the aortic arch, ascending aorta, and the proximal segment of the descending aorta (Fig. 1C). The MaBAPN group displayed an AAD incidence of 80% and a rupture-related mortality of 45%, whereas the FeBAPN group showed a lower AAD incidence of 58% and substantially reduced rupture-related mortality (11%) (Fig. 1D). Consistent with these findings, descending aortic diameters were significantly larger in male AAD/ Rupture mice than in female AAD/ Rupture mice (Fig. 1E).

### Aortic transcriptomic sequencing analysis in BAPN-induced AD mouse models

**Fig. 2:**
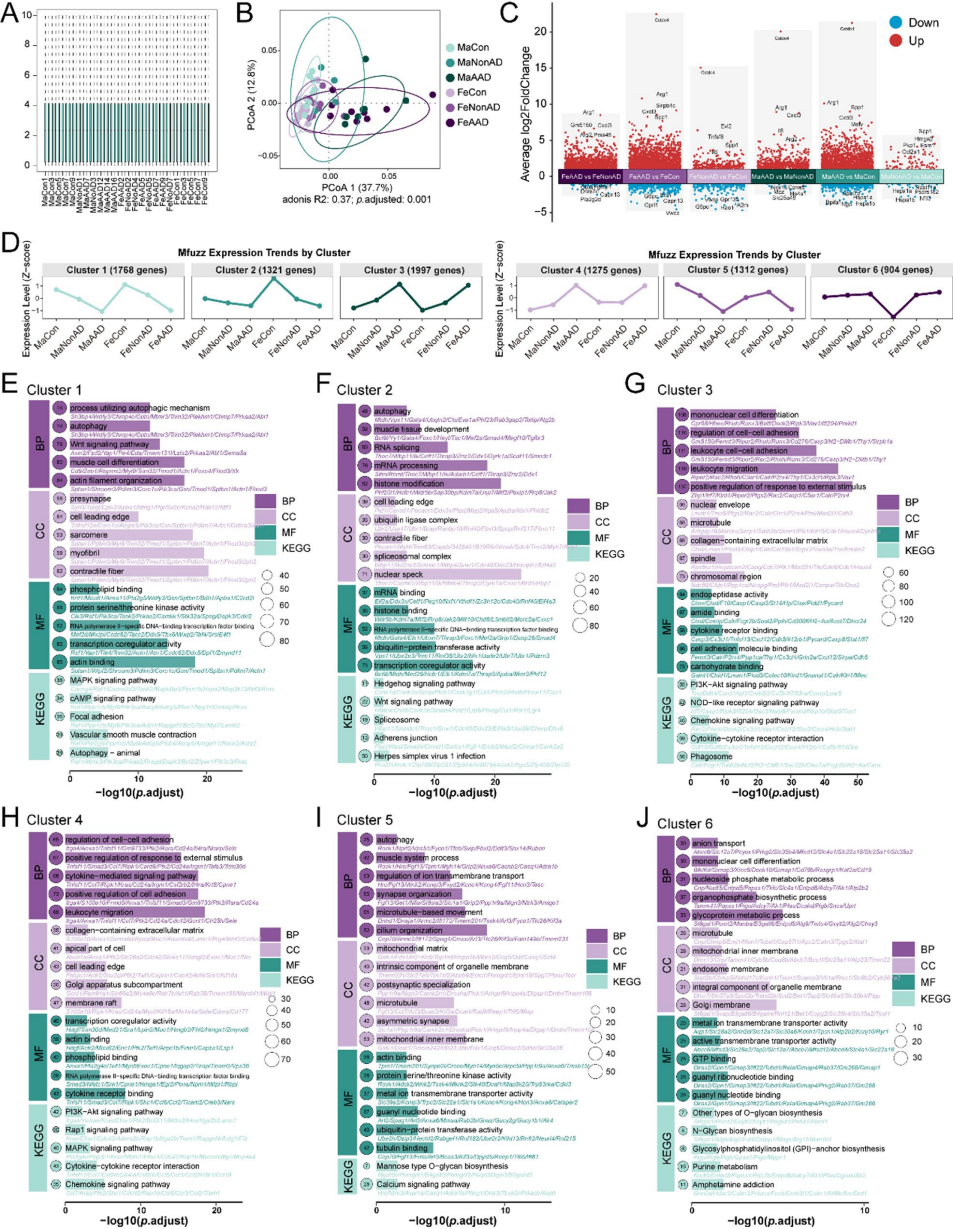
Aortic strand-specific transcriptomic analysis in β-aminopropionitrile (BAPN)-treated mice by sex. (**A**) Box plot of aortic gene expression levels for each sample. (**B**) Principal coordinate analysis (PCoA) plot. (**C**) Volcano plot of multi-group differential expression analysis. (**D**) Pattern clustering analysis plot. (**E-J**) Bar graphs of Gene Ontology (GO) and Kyoto Encyclopedia of Genes and Genomes (KEGG) enrichment analysis for Clusters 1–6, respectively

To characterize sex-related differences in aortic gene expression during AD development, strand-specific RNA-seq of the lesioned thoracic aorta segments from 48 mice was conducted. All the samples were subjected to QC for RNA integrity after extraction. Following sequencing and alignment to the reference genome, the expression matrix was obtained and normalized. The box plot of normalized expression profiles is illustrated in Fig. 2A.

PCoA based on Bray–Curtis distances revealed a clear separation among the Con, NonAD, and AAD groups in both sexes (Fig. 2B). PERMANOVA confirmed significant transcriptomic differences between Con and NonAD, as well as between Con and AAD, for each sex (Table 1). These findings indicate that substantial alterations in gene expression occur before overt AAD formation.

**Table 1:** PERMANOVA and PERMDISP results of pairwise comparisons of transcriptomic profiles across groups

Comparison	R2	*p*-value	Adjusted *p*-value (BH)	Significance	PERMDISP adjusted *p* BH-*p*
FeAAD vs. FeCon	0.34	0.001	0.003	**	0.568
FeAAD vs. FeNonAD	0.23	0.002	0.004	**	0.568
FeNonAD vs. FeCon	0.20	0.002	0.004	**	0.568
MaAAD vs. FeAAD	0.04	0.879	0.879		0.702
MaCon vs. FeCon	0.17	0.001	0.003	**	0.590
MaCon vs. MaAAD	0.39	0.001	0.003	**	0.580
MaCon vs. MaNonAD	0.15	0.025	0.031	*	0.580
MaNonAD vs. FeNonAD	0.15	0.074	0.079		0.955
MaNonAD vs. MaAAD	0.24	0.017	0.023	*	0.580

PERMANOVA, Permutational Multivariate Analysis of Variance. PERMDISP, Permutation test for homogeneity of multivariate dispersions. The R² value represents the effect size (the proportion of variance explained by the group factor). Significance: * adjusted *p*-value < 0.05, ** adjusted *p*-value < 0.01. Abbreviations: Con, Control; AAD, Aortic Dissection or Aneurysm; NonAD, non- aortic dissection or aneurysm; Fe, Female; Ma, Male; BH, Benjamini–Hochberg correction

Differential expression analysis using DESeq2 identified distinct sets of differentially expressed genes (DEGs) across the comparisons (Fig. 2C). Pattern clustering analysis subsequently grouped all DEGs into six expression clusters (Fig. 2D). In Cluster 1, the genes showed a decreasing trend from Con to NonAD to AAD in both males and females and were enriched in pathways associated with vascular smooth muscle cell function, including Wnt signaling, muscle cell differentiation, myofibril and contractile fiber organization, and autophagy-related processes (Fig. 2E). Cluster 2 displayed a similar downward trend but with higher baseline expression in the female controls. These genes were enriched in pathways related to smooth muscle biology, RNA splicing, and histone modification (Fig. 2F). Cluster 3 exhibited a consistent increase from Con to NonAD to AAD in both sexes, and enrichment analysis revealed a predominance of immune-related pathways, particularly leukocyte chemotaxis, cytokine secretion, and inflammation (Fig. 2G). Cluster 4 also showed an overall upward trend, although no marked increase was observed in the FeNonAD group. These genes were again linked to leukocyte chemotaxis and inflammatory signaling (Fig. 2H). Cluster 5 demonstrated a sex-dependent expression pattern, characterized by a progressive decline in males but a transient upregulation in FeNonAD, followed by a reduced expression in FeAAD. Functional analysis associated this cluster with the regulation of autophagy, smooth muscle processes, and mitochondrial function (Fig. 2I). Cluster 6 showed low expression specifically in the female control group, with its functions primarily associated with metal ion transport and related processes (Fig. 2J).

### Transcriptional regulatory analysis and immune cell infiltration analysis

**Fig. 3:**
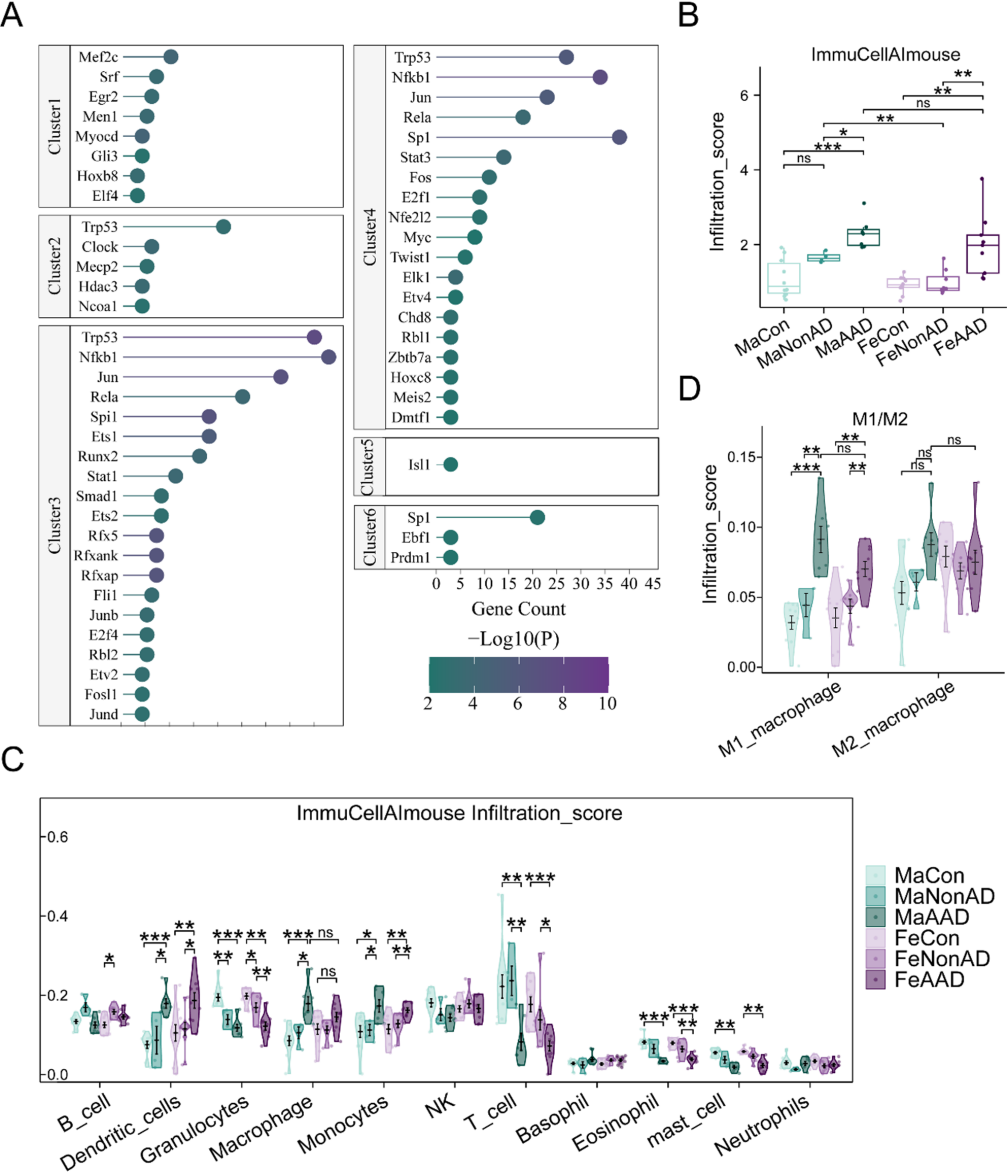
Transcription factor prediction and immune-infiltration analysis in aortas of β-aminopropionitrile (BAPN)-treated mice by sex. (**A**) Transcription factor prediction for each expression cluster. Immune-infiltration scores derived from the Immune Cell Abundance Identifier (ImmuCellAI), including total (**B**), major immune cell types (**C**), and macrophage subtypes (**D**). * *p* < 0.05, ** *p* < 0.01, *** *p* < 0.001

Transcriptional regulatory analysis using the Transcriptional Regulatory Relationships Unraveled by Sentence-based Text mining database revealed potential regulatory transcription factors (TFs) for each cluster. For Cluster 1, Mef2c, Srf, and Egr2 were identified as potential regulators; for Cluster 2, Trp53, Clock, and Mecp2 were identified as potential regulatory TFs. For Clusters 3 and 4, the primary TFs identified were Trp53, Nfkb1, and Jun. For Cluster 5, only Isl1 was identified as a potential regulator. For Cluster 6, Sp1, Ebf1, and Prdm1 were identified as potential regulatory TFs (Fig. 3A).

The overall immune cell infiltration score was significantly increased in both MaAAD and FeAAD groups. Although the MaNonAD group showed an increasing trend, this was not statistically significant; however, its score was significantly higher than that of the FeNonAD group. (Fig. 3B). Among major immune cell types, myeloid populations—including dendritic cells, monocytes, and macrophages—showed increasing trends, whereas granulocytes and T cells tended to decrease with disease progression. Macrophage infiltration was lower in the FeAAD group than in the MaAAD group, although the difference did not reach statistical significance (Fig. 3C). Further analysis of the immune cell subtypes showed that both M1 and M2 macrophages were elevated in AAD, with a statistically significant increase in M1. Although the M1 macrophage score was lower in the FeAAD group than in the MaAAD group, this difference did not reach statistical significance (Fig. 3D).

### Serum untargeted metabolomic analysis in BAPN-induced AD mouse models across sexes

**Fig. 4:**
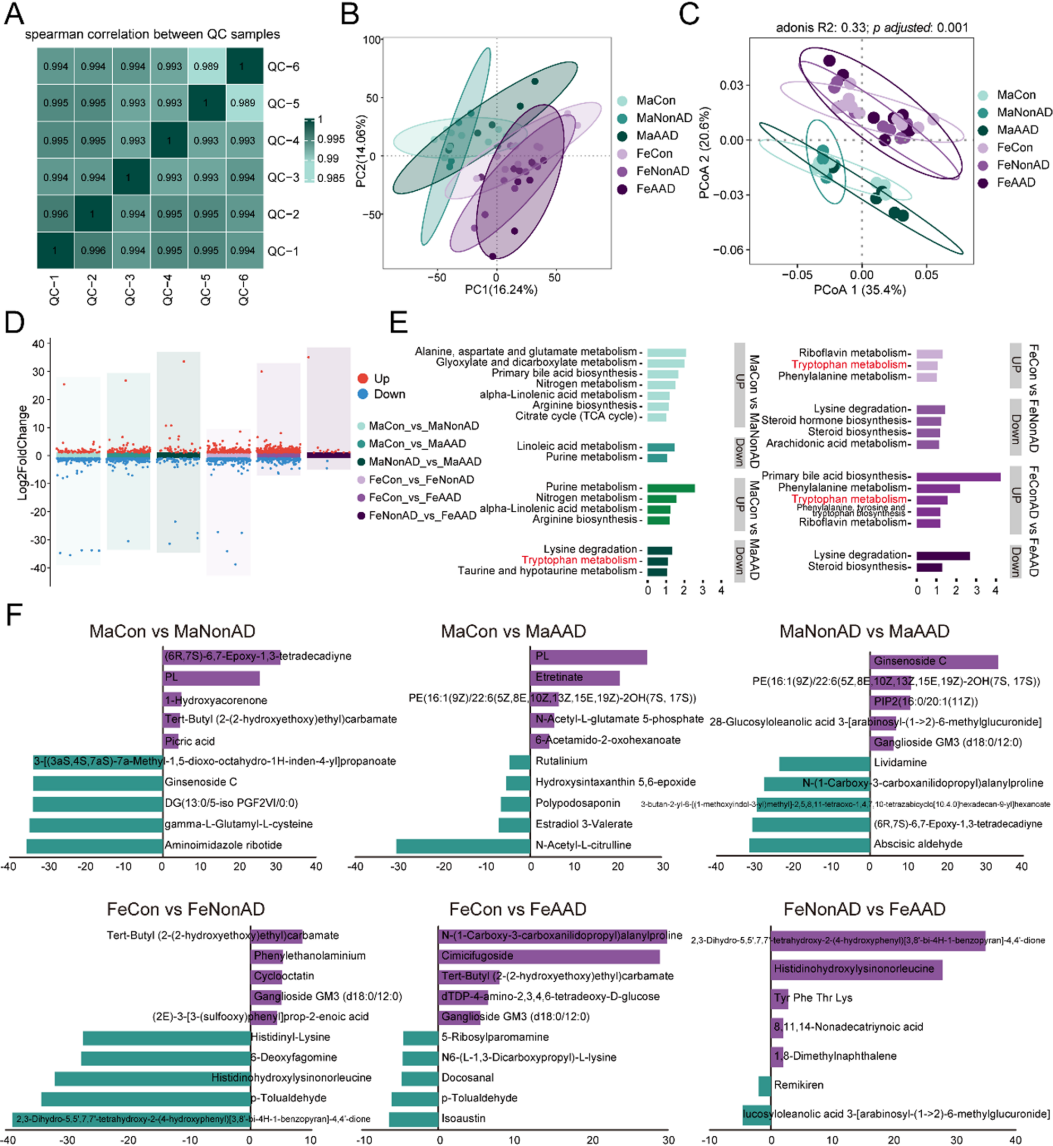
Serum metabolomic analysis of β-aminopropionitrile (BAPN)-treated mice by sex. (**A**) Sample quality control correlation heatmap. (**B**) Principal component analysis plot of different groups. (**C**) Principal coordinate analysis (PCoA) plot of different groups. (**D**) Volcano plot of multi-group differential analysis. (**E**) Kyoto Encyclopedia of Genes and Genomes (KEGG) enrichment plot of differential metabolites from each comparison group. (**F**) Top five upregulated and downregulated metabolites (by logFC) for each comparison group

Untargeted metabolomic profiling of peripheral serum samples identified 5,524 annotated metabolites. QC assessment demonstrated high reproducibility among technical replicates, with correlation coefficients exceeding 0.8 for all QC samples, confirming the robustness of the dataset (Fig. 4A). Principal component analysis revealed distinct separation among the Con, NonAD, and AAD metabolic profiles in both sexes (Fig. 4B). Subsequent PCoA combined with PERMANOVA indicated no statistically significant separation among the MaCon, MaNonAD, and MaAAD groups. In contrast, significant metabolic differences were observed between the FeCon vs. FeNonAD and FeCon vs. FeAAD comparisons, although the FeNonAD and FeAAD groups remained metabolically similar (Fig. 4C; Table 2). These findings suggest that BAPN induces more pronounced systemic metabolic alterations in female mice, and these changes may arise before overt pathological manifestations.

**Table 2:** PERMANOVA and PERMDISP results of pairwise comparisons of serum metabolomic profiles across groups

Comparison	R2	*p*-value	Adjusted *p*-value(BH)	Significance	PERMDISP adjusted *p* BH-*p*
FeAAD vs. FeCon	0.14	0.013	0.020	*	0.508
FeAAD vs. FeNonAD	0.08	0.284	0.304		0.495
FeAAD vs. MaAAD	0.20	0.001	0.003	**	0.508
FeCon vs. FeNonAD	0.16	0.013	0.020	*	0.495
FeCon vs. MaCon	0.32	0.001	0.003	**	0.765
FeNonAD vs. MaNonAD	0.23	0.004	0.009	**	0.508
MaAAD vs. MaCon	0.11	0.065	0.075		0.932
MaAAD vs. MaNonAD	0.11	0.348	0.348		0.765
MaCon vs. MaNonAD	0.13	0.053	0.066		0.765

PERMANOVA, Permutational Multivariate Analysis of Variance. PERMDISP, Permutation test for homogeneity of multivariate dispersions. The R² value represents the effect size (the proportion of variance explained by the group factor). Significance: * adjusted *p*-value < 0.05, ** adjusted *p*-value < 0.01. Abbreviations: Con, Control; AAD, Aortic Dissection or Aneurysm; NonAD, non- aortic dissection or aneurysm; Fe, Female; Ma, Male; BH, Benjamini–Hochberg correction

Differential metabolite analysis identified significantly altered metabolites across group comparisons, as illustrated in the volcano plots (Fig. 4D). KEGG enrichment analysis showed that tryptophan metabolism was significantly downregulated in MaAAD but upregulated in both the FeNonAD and FeAAD groups (Fig. 4E). The top five upregulated and downregulated metabolites based on the fold change for each comparison are illustrated in Fig. 4F.

**Fig. 5:**
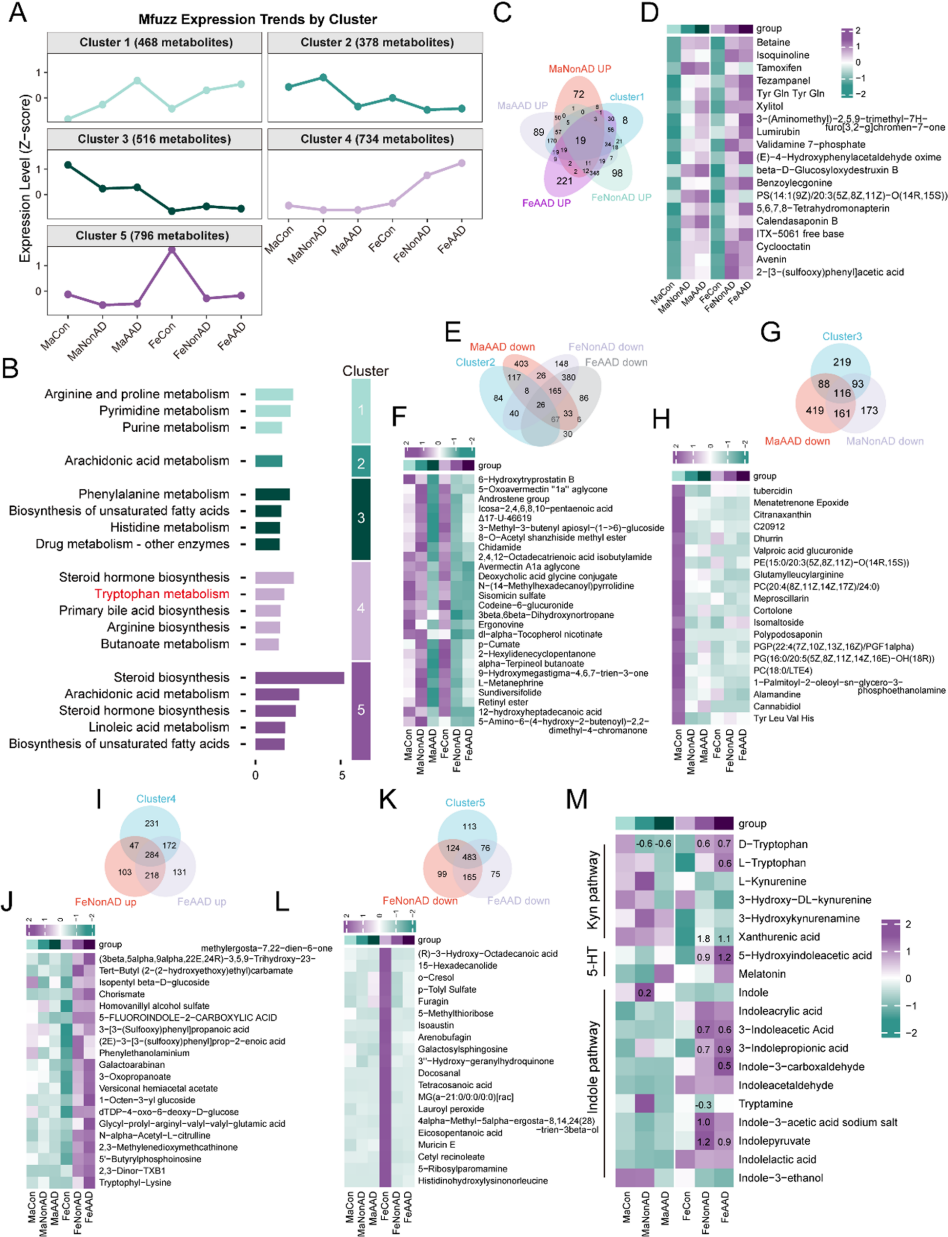
Cluster-based analysis of serum metabolites in β-aminopropionitrile (BAPN)-treated mice by sex. (**A**) Pattern clustering analysis of differentially expressed metabolites. (**B**) Kyoto Encyclopedia of Genes and Genomes (KEGG) enrichment analysis of metabolites for each cluster. (**C** and **D**) Venn diagram and heatmap of 19 intersecting Cluster 1 metabolites upregulated across all BAPN-treated groups. (**E** and **F**) Venn diagram and heatmap of 26 intersecting Cluster 2 downregulated in male AD (MaAAD), female non-aortic dissection (FeNonAD), and female AD (FeAAD) groups. (**G** and **H**) Venn diagram and heatmap of the top 20 Cluster 3 metabolites downregulated in MaAAD and MaNonAD groups. (**I** and **J**) Venn diagram and heatmap of the top 20 Cluster 4 metabolites upregulated in FeNonAD and FeAAD groups based on average logFC. (**K** and **L**) Venn diagram heatmap of the top 20 Cluster 5 metabolites downregulated in FeNonAD and FeAAD groups, ranked by average logFC. (**M**) Heatmap of tryptophan metabolite expression levels

Pattern clustering analysis on all differential metabolites identified five distinct metabolite clusters with characteristic expression patterns (Fig. 5A and B). Cluster 1 metabolites showed a progressive increase from Con to NonAD to AAD, primarily enriched in arginine and proline metabolism, pyrimidine metabolism, and purine metabolism. Nineteen metabolites within this cluster were significantly upregulated in all BAPN-treated groups (Fig. 5C and D). Cluster 2 exhibited decreased levels in AAD and was predominantly enriched in arachidonic acid metabolism, with 26 metabolites significantly altered across all groups, all showing reduced abundance (Fig. 5E, F). Cluster 3 displayed a male-specific decreasing trend from Con to NonAD to AAD and was enriched in phenylalanine metabolism and the biosynthesis of unsaturated fatty acids. The top 20 metabolites ranked by average log fold change are shown in Fig. 5G and H. Cluster 4 demonstrated female-specific elevation after BAPN induction and was enriched in steroid hormone biosynthesis, tryptophan metabolism, primary bile acid biosynthesis, arginine biosynthesis, and butanoate metabolism. The top 20 upregulated metabolites in this cluster are shown in Fig. 5I and J. Cluster 5 was characterized by enrichment in the FeCon group, with pathways including steroid biosynthesis and arachidonic acid metabolism; the top 20 metabolites showing significant decreases in both female BAPN-treated groups are presented in Fig. 5K and L.

Given the prominent alterations in tryptophan metabolic pathways in female-specific metabolic signatures, the expression patterns of intermediate metabolites across these pathways were examined. Our analysis revealed sex-dependent alterations in tryptophan metabolism following BAPN induction. Several indole derivatives—including indolepyruvate, indole-3-acetic acid sodium salt, 3-indolepropionic acid, and 3-indoleacetic acid—were significantly elevated only in female mice (Fig. 5M).

### Fecal 16 S rRNA full-length sequencing in BAPN-induced AD mouse models across sexes

**Fig. 6:**
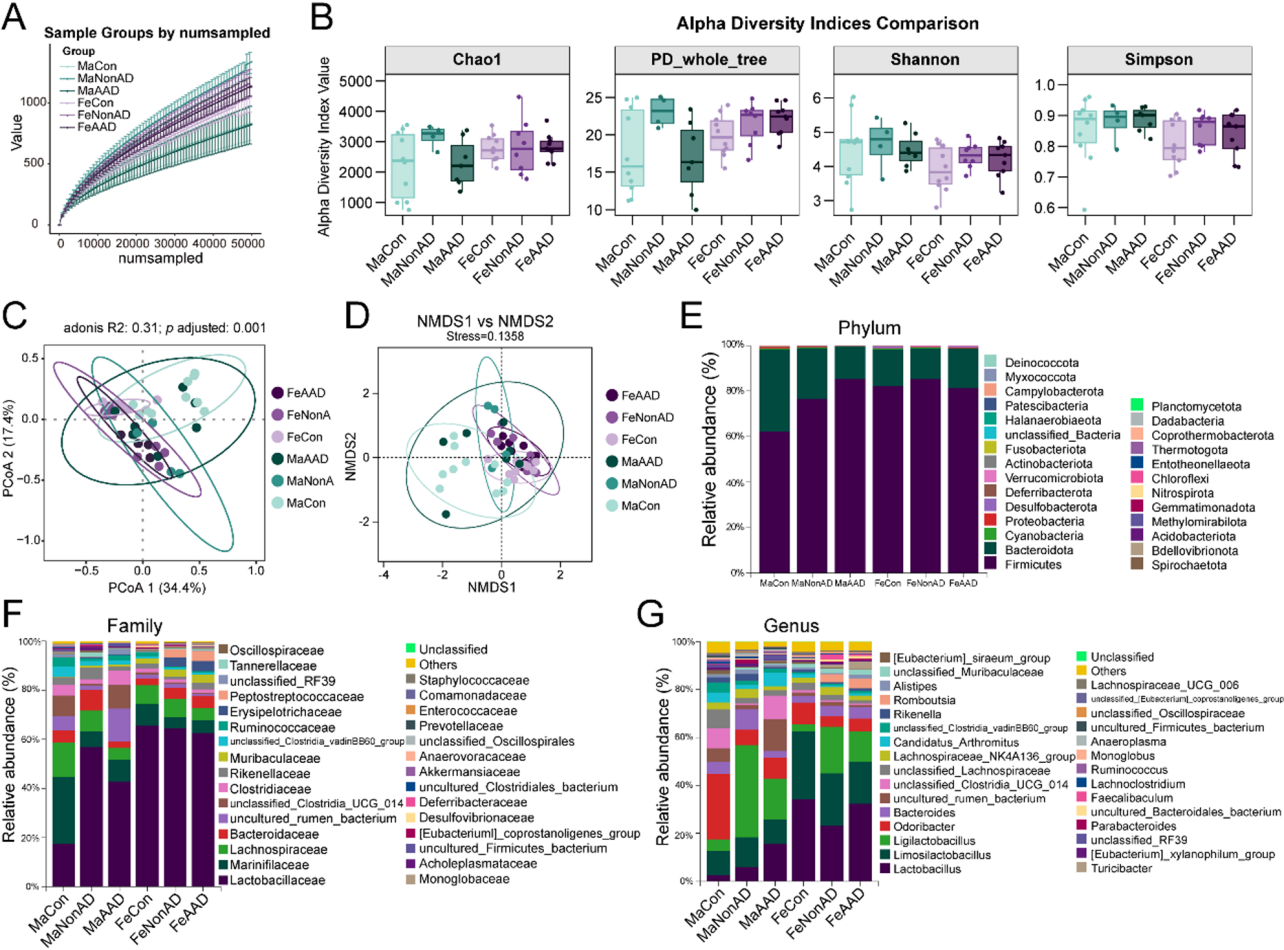
Analysis of gut microbiome in β-aminopropionitrile (BAPN)-treated mice across sexes. (**A**) Rarefaction curves. (**B**) Comparison of alpha diversity indices (Chao1, PD_whole_tree, Shannon, Simpson). (**C**) Principal coordinates analysis (PCoA) and (**D**) non-metric multidimensional scaling (NMDS) plots illustrating the beta-diversity and structural dissimilarity of microbial communities across groups. Stacked bar chart illustrating gut microbiome distribution at the phylum (**E**), family (**F**), and genus (**G**) levels

At the 4-week endpoint, fecal samples from all 48 mice were collected for DNA extraction and full-length 16 S rRNA gene sequencing. The rarefaction curves reached saturation across all samples, confirming adequate sequencing depth (Fig. 6A). Analysis of the alpha diversity ((Chao1, PD_whole_tree, Shannon, Simpson)) based on OTUs revealed no significant differences among the groups (Fig. 6B). In contrast, beta-diversity assessment using Bray–Curtis distances revealed significant differences in microbial community structure between the MaNonAD and MaCon groups and between the FeAAD and FeCon groups by PCoA and PERMANOVA (Fig. 6C; Table 3). Non-metric multidimensional scaling (NMDS) analysis further supported these distinctions (Fig. 6D). Weighted UniFrac analysis yielded pairwise results consistent with those obtained using Bray–Curtis distances, further supporting the robustness of these findings (Figure S2A, Supplementary Table 1). Unweighted UniFrac analysis produced broadly similar patterns, although results should be interpreted with caution given the sensitivity of this metric to rare taxa (Figure S2B, Supplementary Table 2).

**Table 3:** PERMANOVA and PERMDISP results of pairwise comparisons of fecal microbial profiles across groups

Comparison	R2	*p*-value	Adjusted *p*-value(BH)	significance	PERMDISP adjusted *p* BH-*p*
FeAAD vs. FeCon	0.15	0.011	0.028	*	0.407
FeAAD vs. FeNonAD	0.04	0.696	0.696		0.099
FeAAD vs. MaAAD	0.16	0.021	0.038	*	0.067
FeCon vs. FeNonAD	0.14	0.039	0.059		0.084
FeCon vs. MaCon	0.31	0.001	0.005	**	0.007
FeNonAD vs. MaNonAD	0.16	0.135	0.156		0.635
MaAAD vs. MaCon	0.12	0.044	0.060		0.988
MaAAD vs. MaNonAD	0.12	0.239	0.256		0.345
MaCon vs. MaNonAD	0.19	0.023	0.038	*	0.126

PERMANOVA, Permutational Multivariate Analysis of Variance. PERMDISP, Permutation test for homogeneity of multivariate dispersions. The R² value represents the effect size (the proportion of variance explained by the group factor). Significance: * adjusted *p*-value < 0.05, ** adjusted *p*-value < 0.01. Abbreviations: Con, Control; AAD, Aortic Dissection or Aneurysm; NonAD, non- aortic dissection or aneurysm; Fe, Female; Ma, Male; BH, Benjamini–Hochberg correction

**Fig. 7:**
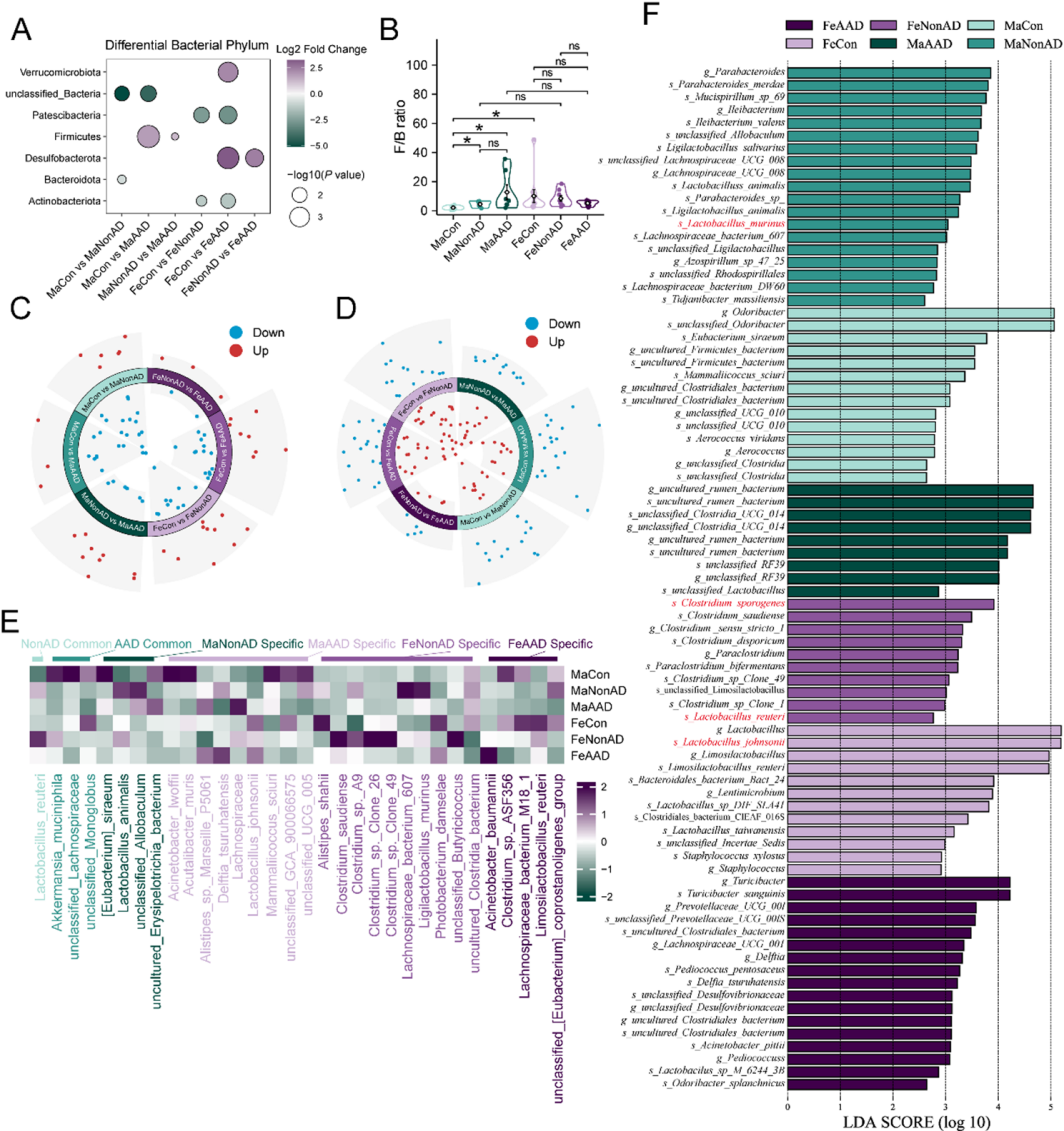
Differential gut microbiota analysis. (**A**) Dot plot displaying differentially abundant phyla at the phylum level. (**B**) Violin plot illustrating Firmicutes/Bacteroidota (F/B) ratio statistics. Volcano plots of multi-group differential analysis at the genus (**C**) and (**D**) species levels. (**E**) Heatmap illustrating expression levels of shared or group-specific differentially abundant bacterial species across comparison groups. (**F**) Bar chart of linear discriminant analysis scores from Linear Discriminant Analysis Effect Size (LEfSe) analysis. * *p* < 0.05

Microbial composition analysis revealed distinct profiles across taxonomic levels (Fig. 6E–G). Differential abundance analysis using DESeq2 identified significantly altered taxa. At the phylum level, compared to the MaCon group, the MaNonAD group showed a significant reduction in Bacteroidota, whereas the MaAAD group showed an increase in Firmicutes. In females, the FeNonAD group displayed reduced Actinobacteria and Patescibacteria compared to the FeCon group, whereas the FeAAD group showed increased Desulfobacterota and Verrucomicrobiota along with reduced Actinobacteria and Patescibacteria relative to the FeCon group (Fig. 7A). The Firmicutes/Bacteroidota (F/B) ratio was significantly elevated in male NonAD and AAD mice (Fig. 7B). Volcano plots highlighted the genus- and species-level differences (Figs. 7C and D). Species-level UpSet plot analysis revealed increased *Lactobacillus reuteri* in the NonAD groups of both sexes, whereas *unclassified Lachnospiraceae*, *Akkermansia muciniphila*, and *unclassified Monoglobus* were differentially enriched in the AAD groups. Notably, *Akkermansia muciniphila* decreased in MaAAD but increased in FeAAD (Fig. 7E).

LDA effect size (LEfSe) identified the key taxa distinguishing the experimental groups (Fig. 7F). Species capable of metabolizing tryptophan into indole derivatives—such as *Lactobacillus reuteri* and *Clostridium sporogenes*—were significantly enriched in the FeNonAD group. In contrast, *Lactobacillus johnsonii* was enriched in the FeCon group, and *Lactobacillus murinus* was characteristic of the MaNonAD group. To ensure the robustness of these findings, the sequencing data were independently re-analyzed using the high-resolution DADA2 pipeline. This approach confirmed a similar separation of microbial communities to the OTU-based results, and the representative major taxa at the genus and species level exhibited high consistency across groups (Figure S2C, D). Notably, LEfSe analysis identified the genus *Clostridium_sensu_stricto_1*, along with the species *Clostridium disporicum* and *Clostridium sp. Clone_1*, as characteristic taxa robustly enriched in the FeNonAD group (Figure S2D).

**Fig. 8:**
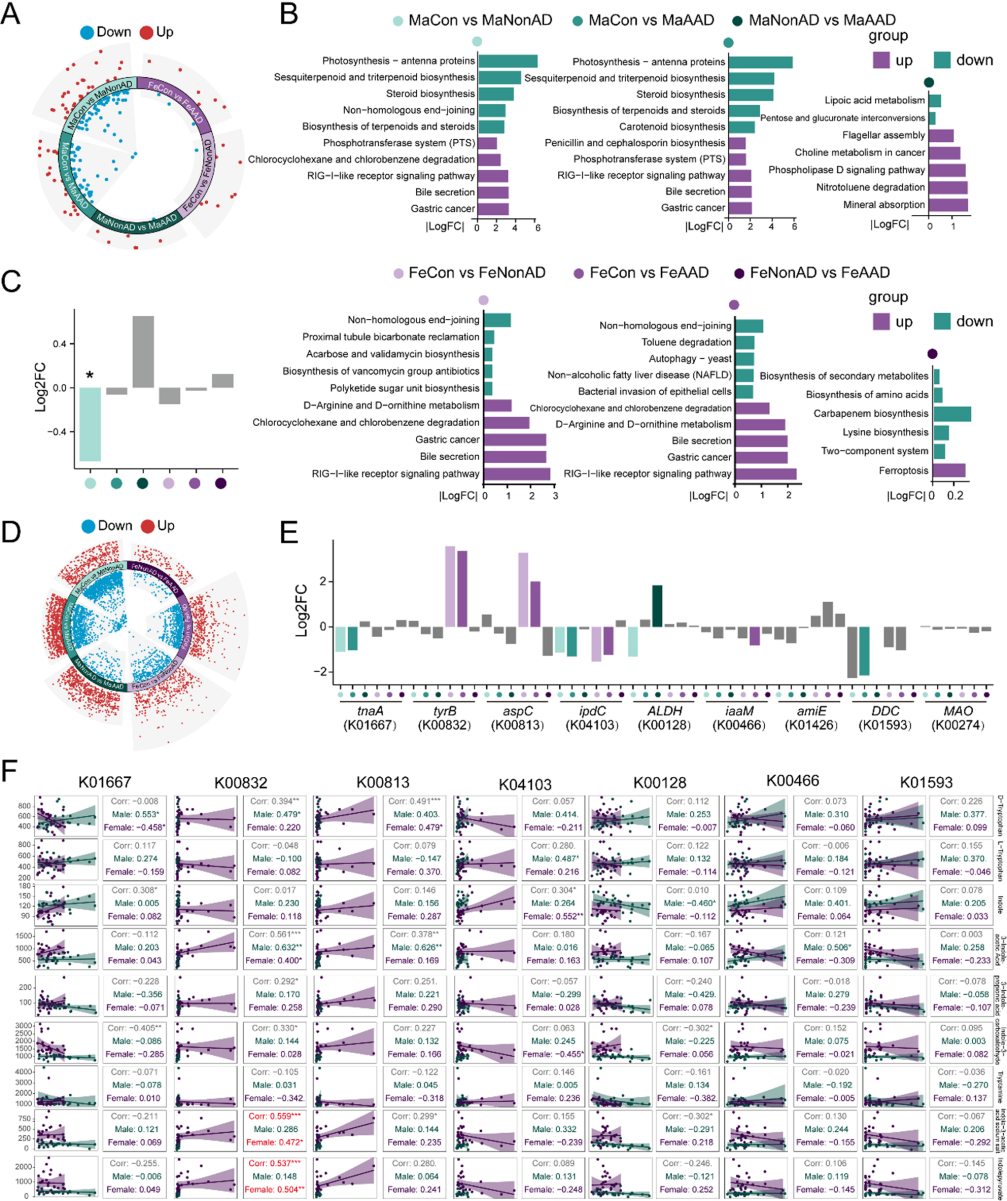
Functional prediction of gut microbiota using PICRUSt2 (Phylogenetic Investigation of Communities by Reconstruction of Unobserved States 2). (**A**) Volcano plots of multi-group differential analysis at Kyoto Encyclopedia of Genes and Genomes (KEGG) Level 3. (**B**) Top five upregulated and downregulated Level 3 pathways across comparison groups. (**C**) Bar chart illustrating logFC values of the “Tryptophan metabolism” pathway across groups. (**D**) Volcano plots of multi-group differential analysis at the KEGG orthology (KO) level. (**E**) Bar chart illustrating logFC values of enzymes involved in the tryptophan–indole pathway across groups. (**F**) Correlation heatmap between these enzymes and differentially expressed tryptophan–indole pathway metabolites

To evaluate the functional metabolic capacity, PICRUSt2 was used to infer microbial gene pathways, followed by differential KEGG Level 3 and enzyme-level analyses (Fig. 8A and B). Tryptophan metabolism was significantly reduced in the MaNonAD group but unchanged in other comparisons (Fig. 8C). Enzyme-level analysis demonstrated significant upregulation of *tyrB* and *aspC* and downregulation of *ipdC* in both the FeNonAD and FeAAD groups (Fig. 8D, E). In females, *tyrB* expression correlated positively with indolepyruvate and indole-3-acetic acid sodium salt (Fig. 8F). Importantly, PICRUSt2 functional predictions based on ASVs similarly verified the significant upregulation of *tyrB* and *aspC* in the FeNonAD and FeAAD groups (Figure S2E).

### Integrated multi-omics analysis

**Fig. 9:**
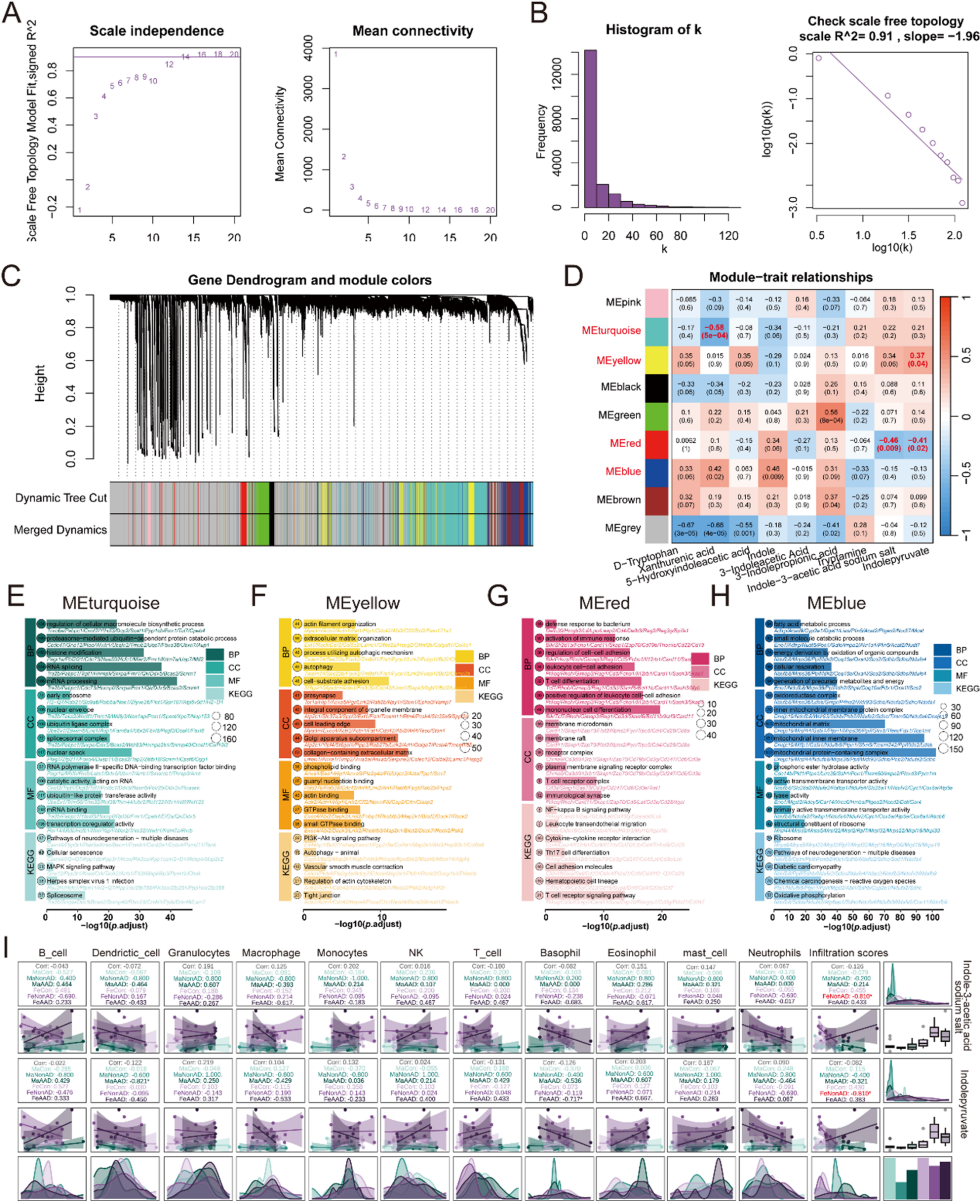
Multi-omics analysis of the tryptophan–indole metabolic pathway. (**A**) Selection of soft-thresholding power (β) for scale-free network construction. (**B**) Histogram of node connectivity (k) and a scatter plot of log10(p(k)) versus log10(k) confirm the scale-free property (R² = 0.91). (**C**) Cluster dendrogram of co-expression modules identified by weighted gene co-expression network analysis. (**D**) Gene Ontology (GO) and Kyoto Encyclopedia of Genes and Genomes (KEGG) functional enrichment analysis of hub genes within the turquoise (**E**), yellow (**F**), red (**G**), and blue (**H**) co-expression modules. Bar length represents the enrichment significance expressed as −log10(adjusted *p*-value). Only terms with adjusted *p* < 0.05 are shown. (**I**) Correlation heatmap between indolepyruvate and indole-3-acetate sodium salt levels with immune-infiltration scores

To investigate potential mechanisms by which indole metabolites exert protective effects before dissection, we performed WGCNA using transcriptomic data from MaCon, MaNonAD, FeCon, and FeNonAD samples. Scale-independence assessment indicated that a soft-threshold power of β = 14 yielded a scale-free topology fit (R² > 0.9) whereas maintaining a high mean connectivity (Fig. 9A). The scale-free topology fit plot further showed a strong linear relationship between log(k) and log(p(k)) at this threshold, fulfilling the criteria for scale-free network construction (Fig. 9B). Using dynamic tree cutting followed by module merging, nine distinct gene modules were identified, excluding the unassigned gray module from downstream analyses (Fig. 9C).

To explore the relationships between indole metabolites and gene co-expression modules, Spearman correlation coefficients were calculated, and the module-trait relationships were visualized (Fig. 9D). The turquoise module showed a significant negative correlation with xanthurenic acid. The yellow module was positively correlated with indolepyruvate. The red module showed significant negative correlations with both indolepyruvate and indole-3-acetic acid sodium salt, whereas the blue module showed positive correlations with xanthurenic acid and indole.

Functional enrichment of these modules revealed biologically coherent pathways (Fig. 9E–H). The turquoise module was enriched in RNA splicing and histone modification (Fig. 9E). The yellow module was enriched in autophagy and smooth muscle contraction pathways (Fig. 9F). The red module was predominantly associated with immune-related processes, including leukocyte adhesion, monocyte and T-cell differentiation, and inflammatory signaling (Fig. 9G). The blue module was enriched in energy metabolism pathways, including cellular respiration and oxidative phosphorylation (Fig. 9H).

Given the significant negative correlations between indolepyruvate/indole-3-acetic acid sodium salt and the immune-related red module, which suggest their potential involvement in modulating immune cell infiltration, their associations with immune-infiltration scores were therefore examined. In the FeNonAD group, both metabolites showed significant negative correlations with the total immune-infiltration score (Fig. 9I).

### Indolepyruvate attenuates BAPN-induced AD in male mice

**Fig. 10:**
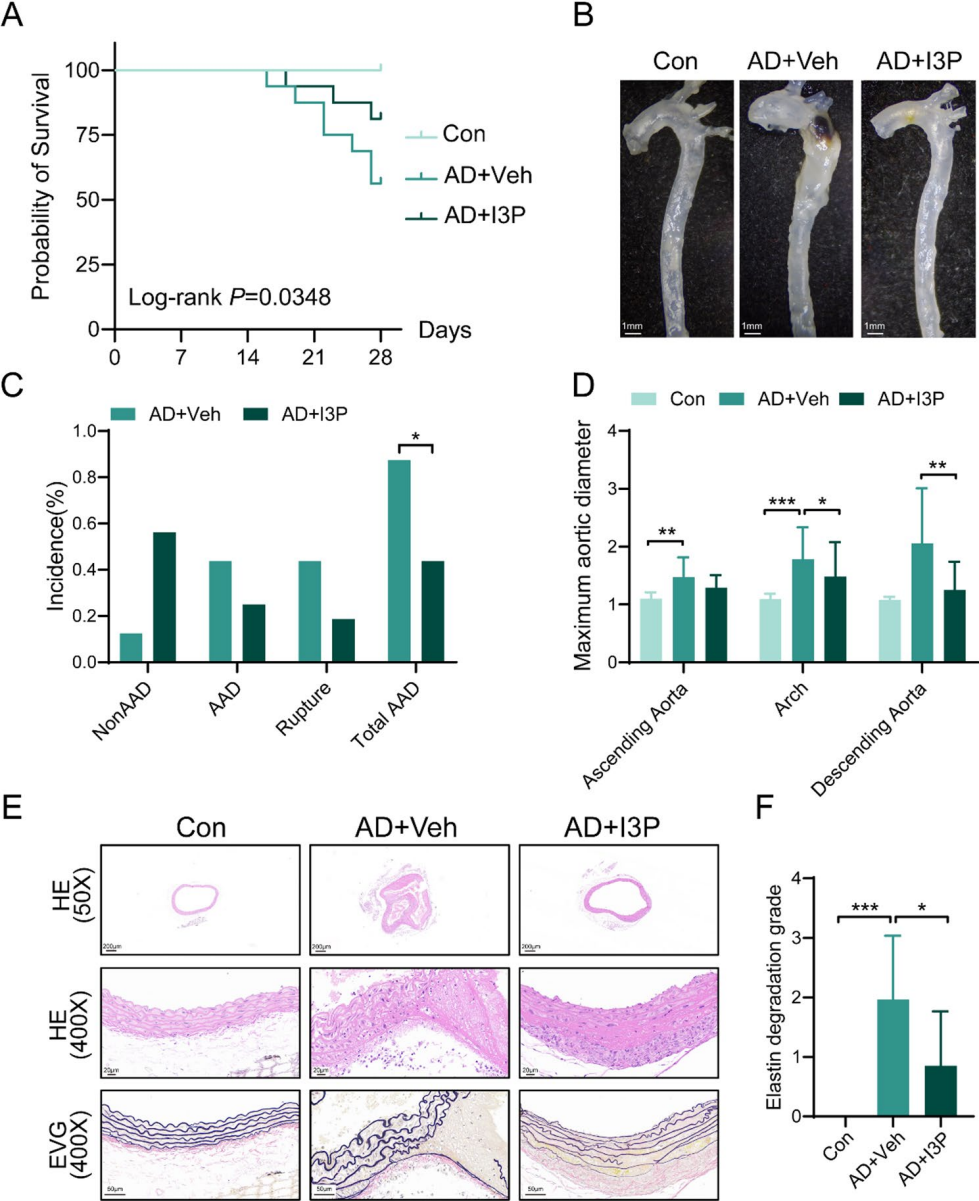
I3P protects against β-aminopropionitrile (BAPN)-induced aortic dissection and medial degradation in male mice. (**A**) Survival curves of mice across different experimental groups; (**B**) Representative gross images of the aorta from each group; (**C**) Statistical analysis of the incidence of aortic lesions in each group; (**D**) Segmental measurements of aortic diameters (ascending aorta, aortic arch, and descending aorta); (**E**) Representative histological images (H&E and EVG staining) of the aorta from each group; (**F**) Quantitative analysis of the elastic fiber degradation score shown as a bar graph. Data are presented as mean ± SEM. *p < 0.05, **p < 0.01, ***p < 0.001

By performing gross morphological imaging, incidence analysis, and segmental diameter measurements of the aortas across all groups, we observed that intraperitoneal injection of I3P significantly improved the survival rate of male mice (Fig. 10A). Furthermore, treatment with I3P notably reduced the overall incidence of AAD in male mice, accompanied by a significant decrease in the diameters of the aortic arch and descending aorta (Fig. 10B–D). Consistent with these morphological improvements, EVG staining and quantitative analysis revealed that I3P treatment significantly lowered the elastic fiber degradation score in the aortic media of male mice (Fig. 10E–F). Taken together, these findings demonstrate that I3P exerts a potent protective effect against AD in males.

## Discussion

Female sex is widely recognized to confer a protective effect against a broad spectrum of cardiovascular diseases. Accordingly, epidemiological data consistently demonstrate a significantly lower incidence of aortic dissection (AD) in women compared to men; however, the precise molecular mechanisms driving this sexual dimorphism remain largely unexplored. In preclinical settings, the female protective advantage has been well-documented in experimental models of abdominal aortic aneurysm (AAA) and Ang II-induced dissecting aneurysms [29, 30]. Nevertheless, there are relatively few studies exploring the BAPN-induced AD model in female animals. Our present study bridges this gap by demonstrating that female mice also exhibit resistance to BAPN-induced AD. Furthermore, this investigation represents the inaugural comprehensive profiling of sex-associated differences in AD within a multi-omics context.

Transcriptomic analysis revealed substantial alterations in aortic gene expression in the NonAD group, even before dissection formation. Pattern clustering analysis revealed that genes in Clusters 1 and 3 changed progressively from Con to NonAD and ultimately to the AAD state in both sexes. Cluster 1 genes were primarily associated with smooth muscle cell function, whereas Cluster 3 genes were enriched for pathways related to immune cell adhesion, cytokine interactions, and inflammatory response. These findings are consistent with prior work demonstrating progressive smooth muscle dysfunction and immune cell infiltration/activation during AAD development in male mice [31]. Our results confirm that similar processes occur during AAD development in females, suggesting that the NonAD state represents an intermediate transitional phase between physiological (Con) and pathological (AAD) conditions. Enrichment analyses of Clusters 2 and 5 revealed that female control mice exhibited higher basal expression of autophagy-related pathway genes and that certain autophagy genes were further upregulated in the FeNonAD group. This pattern aligns with evidence that sex hormones modulate autophagy [32]. Conversely, the enrichment pattern of Cluster 4 indicated that aortas from the FeNonAD mice were resistant to BAPN-induced upregulation of certain inflammation-related genes. Immune-infiltration analysis supported these findings, revealing significantly lower overall immune cell infiltration scores in the FeNonAD mice. Overall, our results indicate that the FeNonAD state is characterized by attenuated immune activation and inflammatory signaling.

Serum metabolomic analysis revealed that female mice exhibited more pronounced alterations in their serum metabolic profiles than males. Notably, changes in metabolic profiles emerged even in the absence of overt aortic pathology (NonAD group). Notably, the serum metabolic profiles of the FeNonAD and FeAAD groups were highly similar, indicating that systemic metabolic alterations in female mice precede local structural aortic pathologies. Nineteen DEMs exhibited progressive increases from Con to NonAD to AAD in both sexes, providing potential candidates for early AAD biomarker development.

Among the identified metabolic pathways, tryptophan metabolism emerged as a potentially central mediator of sex-specific responses. Tryptophan is an essential amino acid primarily obtained through dietary intake. It is metabolized into various bioactive compounds via the kynurenine, serotonin, and indole pathways, all of which are implicated in vascular diseases. The kynurenine pathway has been associated with macrophage-driven inflammation and is upregulated in aortic aneurysm tissues [33]. Conversely, deficiency of indoleamine 2,3-dioxygenase 1, a key enzyme metabolizing tryptophan in the aorta, limits the progression of abdominal aortic aneurysm (AAA) induced by Ang II combined with a high-fat diet, highlighting a role for the kynurenine pathway in disease development [34, 35]. The indole pathway of tryptophan metabolism may exert protective effects on vascular tissue. For instance, concentrations of indole-3-propionic acid (IPA) and indole-3-aldehyde correlate inversely with the risk of peripheral artery disease (PAD) [36], whereas decreased levels of indole-3-acetic acid (IAA) and IPA are associated with an increased risk of atherosclerotic cardiovascular disease (ASCVD) [37]. In vivo experiments demonstrated that both indole-3-aldehyde and IPA attenuate the progression of experimental AD [38, 39]. Inherent age-dependent sex differences were found in tryptophan-derived indole metabolites, including indolepyruvate. Levels were comparable between 3-week-old female and male mice; however, they were significantly higher in 53-week-old female mice than in age-matched males [40]. In our study, the levels of the relevant metabolite in female mice were similar to those in male mice before BAPN induction. However, after induction, metabolites of the indole pathway were significantly higher in female mice. Therefore, it was hypothesized that tryptophan metabolism, particularly the indole pathway, may contribute to the divergent susceptibility to BAPN-induced AAD.

To test this hypothesis, we performed an interventional rescue experiment by supplementing indolepyruvate to more susceptible male mice. Our results demonstrated that I3P treatment significantly improved survival rates and preserved aortic structural integrity, effectively mitigating the progression of BAPN-induced injury. These findings provide functional evidence that the enrichment of indole metabolites observed in females acts as a contributor of vascular protection rather than a mere associative marker.

Because the production of indole metabolites from tryptophan depends heavily on gut microbiota, we further investigated microbial composition. Recent studies using germ-free and pseudogerm-free mouse models have implicated the gut microbiota in the pathogenesis of cardiovascular diseases, including atherosclerosis, hypertension, and AAA [41–43]. Microbiota influence cardiovascular disease progression by modulating host metabolism and immune responses. Compositional differences in the gut microbiota of patients with AD and healthy controls were observed, highlighting a potential role for gut microbes in AD pathogenesis [25], although the current body of evidence remains limited. Clinical sample-based studies have reported no statistically significant differences in alpha diversity, yet have consistently observed significant differences in beta diversity between AD and controls [25].

In contrast, recent analyses of BAPN-induced mice have documented reduced alpha diversity in AD groups [44, 45]. Our analysis revealed that, whereas alpha diversity remained unchanged, the F/B ratio was markedly elevated in the male AAD group. This ratio is implicated in enhanced energy metabolism and is a recognized biomarker for obesity and increased cardiovascular disease risk [46–48].

According to the OTU-based analysis at the species level, *Akkermansia muciniphila* exhibited opposite trends between sexes, decreasing in male AAD mice while increasing in female AAD. This species is widely recognized as a probiotic that suppresses the progression of atherosclerosis, AAA, and hypertension [49–51]. Several species in the genus *Clostridium*, including *Clostridium saudiense*, *Clostridium sp. A9*, *Clostridium sp. Clone_26*, and *Clostridium sp. Clone_49* was specifically enriched in the FeNonAD group. Although these species remain largely uncharacterized, the genus *Clostridium* contributes to the synthesis of short-chain fatty acids. Notably, butyrate has been proven to confer protection against AD [45, 52]. Several bacterial species that differentially alter tryptophan metabolism were examined. *Lactobacillus reuteri* was significantly increased in both male and female NonAD groups. Moreover, it was a defining species of the FeNonAD group. *L. reuteri* is a well-established probiotic used for acute diarrhea treatment in children [53]. The transformation of tryptophan by *L. reuteri* generates multiple indole derivatives [54]. *Lactobacillus johnsonii*, which synthesizes indole-3-propionic acid from tryptophan, was significantly elevated in MaAAD mice in our study, although heat map visualization indicated even stronger enrichment in female mice [55]. *Clostridium sporogenes*, also a characteristic species of the FeNonAD group, possesses a complete metabolic pathway for converting tryptophan to indolepropionic acid via indolepyruvate [56]. Further analysis via the more stringent DADA2 pipeline identified fewer differentially abundant taxa than OTU-based analysis, but the results showed high consistency with OTU-derived findings at the genus level. Notably, the genus *Clostridium_sensu_stricto_1* and the species *Clostridium disporicum* were robustly enriched in the FeNonAD group. While research on these specific taxa remains limited, existing evidence suggests they are closely associated with tryptophan metabolism [57, 58].

Given that tryptophan metabolism primarily depends on specific enzymes, PICRUSt2 was used to assess changes in the gut microbiota’s metabolic potential across groups. PICRUSt2-based functional predictions supported a pronounced enhancement of the tryptophan–indole pathway in female mice following BAPN induction. Importantly, these functional predictions demonstrated remarkable consistency across different bioinformatic pipelines. The predicted abundance of genes encoding transaminases *tyrB* and *aspC*, which catalyze the formation of indolepyruvate from tryptophan, was significantly elevated in both the FeNonAD and FeAAD groups [59, 60]. Consistent with these genomic predictions, indolepyruvate levels were markedly increased in the serum of these groups. However, we observed an apparent discrepancy regarding indole-3-acetic acid (IAA) synthesis: although IAA was significantly upregulated in female serum, the predicted abundance of the *ipdC* gene (encoding indolepyruvate decarboxylase) was significantly reduced [61]. To clarify this, we further mined our PICRUSt2 data for alternative IAA biosynthesis routes, including the indole-3-acetamide (IAM) pathway (*iaaM*/K00466 and *amiE*/K01426) and the tryptamine pathway (*DDC*/K01593 and *MAO*/K00274). Interestingly, these alternative routes did not show upregulation. This divergence between predicted gene abundance and observed metabolite levels likely stems from substrate-driven metabolic flux. Our metabolomics data showed that upstream precursors, particularly tryptophan, were robustly maintained or increased in females. According to the principles of enzyme kinetics, high substrate availability can drive an increased metabolic rate even when the concentration of the encoding enzymes remains stable or slightly decreased. Furthermore, the elevated systemic IAA likely reflects the cumulative microbial output and host absorption dynamics integrated over the 28-d induction period, which a cross-sectional prediction at a single timepoint may not fully capture. Finally, it is important to emphasize that PICRUSt2 provides an estimation of the functional potential based on 16 S rRNA sequences, which does not equate to actual in vivo metagenomic gene expression or real-time enzymatic activity.

Integrated multi-omics analysis using WGCNA revealed preliminary links between circulating indole metabolites and aortic gene expression. Both indole-3-acetic acid sodium salt (the chemical form of indole-3-acetic acid in positive ion mode) and indolepyruvate demonstrated significant negative correlations with the red gene module, which was enriched for pathways regulating T-cell/monocyte differentiation, immune cell chemotaxis, and inflammatory responses. Moreover, indolepyruvate showed a significant positive correlation with the yellow gene module, which was enriched in vascular smooth muscle cell function and autophagy pathways. These results strongly suggest that the two indole metabolites may contribute to the female-protective effect by attenuating inflammation and promoting autophagy. This interpretation is consistent with established mechanistic data. Indolepyruvate suppresses lipopolysaccharide (LPS)-induced interleukin 1β (IL-1β) production in macrophages by reducing hypoxia-inducible factor 1-alpha (HIF-1α) levels and glycolysis [62]. IAA inhibits T-cell differentiation into Th17 cells while promoting regulatory T-cell (Treg) development [63].

Mechanistically, indole derivatives, such as indolepyruvate and IAA, are recognized as potent endogenous ligands for the aryl hydrocarbon receptor (AhR), which exerts its biological effects through distinct canonical and non-canonical signaling pathways. The canonical pathway, characterized by AhR-ARNT dimerization and binding to xenobiotic response elements (XRE), primarily mediates the transcription of cytochrome P450 enzymes (e.g., *Cyp1a1*, *Cyp1b1*) for xenobiotic detoxification. In contrast, non-canonical AhR signaling—often independent of classical DNA binding—plays a pivotal role in immune regulation, complementing the canonical pathway [64, 65]. Macrophages lacking AhR hyperproduce IL-1β, IL-6, and tumor necrosis factor-α (TNF-α) upon stimulation, a pro-inflammatory state that drives subsequent infiltration and M1 polarization in vivo [66, 67]. Moreover, AhR activation suppresses Th17 differentiation and promotes Treg development, thereby mitigating inflammatory diseases, such as rheumatoid arthritis and colitis [68, 69]. In AD, Th17/Treg imbalance correlates with disease progression, and lower Th17 levels correlate with improved postoperative vascular remodeling in type B AD [70, 71]. Single-cell sequencing studies have confirmed that inflammatory cell infiltration, predominantly by macrophages, represents a defining pathological feature of AD. Moreover, targeting macrophages or macrophage-related inflammatory factors effectively reduces both the incidence and rupture of experimental AD [31, 72, 73].

Furthermore, these metabolites also serve as ligands for the pregnane X receptor (PXR), which provides an additional layer of vascular protection. Similar to non-canonical AhR signaling, PXR activation antagonizes NF-kappaB-mediated inflammatory responses, particularly by reducing the expression of adhesion molecules such as ICAM1 and VCAM1 [74, 75]. This aligns with our observation of lower inflammatory cell recruitment and attenuated vascular remodeling in female mice. Notably, given that PXR activation has been shown to suppress the progression of AAA, it may also exert protective effects against aortic dissection [76].

Beyond immune regulation, AhR plays a direct role in modulating autophagy; its activation by endogenous ligands enhances autophagy by increasing the transcription of BCL2-interacting protein 3, thereby maintaining mitochondrial homeostasis in hepatocytes [77], and it can directly bind to the PINK1 promoter to enhance PINK1/Parkin-mediated mitophagy, thereby reducing LPS-induced cellular inflammation [78]. Autophagy has an established role in protecting smooth muscle cells against AAD formation. The autophagy activator rapamycin exerted inhibitory effects across multiple AD models, including BAPN-induced AD [79–82]. Therefore, specific enrichment of indolepyruvate and IAA in female mice may contribute to AD protection by serving as AhR ligands that jointly restrain immune inflammation and promote autophagic homeostasis.

In summary, integrated multi-omics analysis in this study revealed a sex-specific activation of the tryptophan–indole metabolic axis in BAPN-induced female AD models and linked this metabolic shift to reduced aortic inflammation. These findings provide novel mechanistic insights into female-associated protection in AD and highlight the gut microbiota–tryptophan–AhR axis as a potential target for sex-informed therapeutic strategies.

Several limitations of this study should be acknowledged. Although the BAPN-induced mouse model effectively recapitulates key pathological hallmarks of human AD, it may not fully capture the complex clinical heterogeneity seen in patients. Although internal standards and rigorous QC procedures were implemented in our untargeted metabolomics workflow, targeted metabolomics with authentic standards for the key indole metabolites is still needed to further increase confidence in their identification. In terms of experimental validation, RT-qPCR validation of key sex-differentially expressed genes in aortic tissue — particularly inflammation-related genes—was not performed in the current study due to complete allocation of tissue to RNA-seq library preparation; such validation, along with the development of broadly degenerate primers for community-wide quantification of *tyrB* and *aspC* in fecal DNA, represents important priorities for future investigation. Furthermore, the lack of established baseline statistics for AD incidence and rupture-related mortality in female mice in this specific model precluded a formal a priori power calculation, although our sample size was robust enough for high-dimensional omics profiling. Methodologically, the inherent cage effect remains a potential confounder in microbiome research. Because male and female mice were housed separately to avoid pregnancy, sex-specific microbial signatures may be partially influenced by distinct housing environments, even though we mitigated this by sampling across multiple cages and standardizing facility conditions. Additionally, our cross-sectional approach at the 4-week endpoint limits the ability to track the dynamic evolution of the “gut–tryptophan–aorta” axis. Future longitudinal investigations of the prodromal phase—specifically at the 2-week mark or earlier—would be instrumental in determining whether the metabolic shifts observed in females are primary protective drivers that precede overt vascular injury.

A particularly limiting aspect of this study is the specific age window of the animals used (3–7 weeks), which spans the onset of puberty, and the fact that the estrous cycle of female mice was neither monitored nor controlled. Existing evidence demonstrates that estrogen levels can significantly modulate both gut microbiota composition and the systemic immune-inflammatory status [83]. Consequently, fluctuating sex hormones may act as a confounding factor in this study, complicating the interpretation of inter-group differences and likely contributing to the observed variance in the female omics data.

Although sex hormones play a pivotal role in shaping sex differences, their specific involvement in the pathogenesis of AD remains highly complex [15, 84]. Beyond the classical regulation by sex hormones, intrinsic biological differences dictated by sex chromosome complement—such as X-chromosome dosage effects and Y-chromosome-specific gene functions—may also establish distinct baseline metabolic and immune phenotypes between sexes [85, 86]. Our unbiased, integrated multi-omics approach aimed to systematically explore the molecular networks underlying sex differences in AD. The “gut microbiota–tryptophan metabolism–vascular inflammation” axis identified herein may be partially regulated by sex hormones or reflect more deeply ingrained, inherent biological sexual dimorphisms. Future studies employing models that strictly control for sex hormone variables (e.g., via gonadectomy or hormone replacement) will be crucial to disentangle hormone-dependent mechanisms from chromosome-intrinsic factors, thereby precisely elucidating the sexual dimorphism in AD susceptibility.

Finally, although we confirmed that indolepyruvate exerts a protective effect against AD in males, and our broader analyses suggest that modulating inflammation may be a potential pathway for its protective action, the specific immune cell subsets involved and the precise underlying mechanisms remain to be elucidated by future research. Furthermore, exploring whether modulating the gut microbiota can influence AD susceptibility and whether fecal microbiota transplantation could serve as a therapeutic strategy for AD represents promising directions for future studies.

## Conclusions

This study demonstrates that female mice exhibit significantly lower susceptibility to AD in a BAPN-induced model. Metabolites of the tryptophan–indole pathway were selectively elevated in BAPN-treated female mice, likely reflecting sex-specific alterations in the gut microbiota. Notably, supplementation with indolepyruvate suppressed AD progression in male mice, suggesting that the gut microbiota-associated tryptophan–indole metabolic pathway may contribute to the protective effects observed in females.

## Supplementary Information

Supplementary Material 1.

Supplementary Material 2.

## Data Availability

The data supporting the findings of this study are available in the following repositories and within the article’s additional file: the transcriptome datasets generated and analyzed are available in the GEO repository under accession number GSE325608, the 16 S rRNA sequencing data are available in the SRA repository under accession number PRJNA1437302, and the untargeted metabolomics data are provided within the Supplementary Table 3 of this article.
