# Supplementary material for: Integrated multi-omics analysis reveals a gut microbiota–tryptophan metabolism axis contributes to sex differences in a β-aminopropionitrile-induced aortic dissection mouse model
Source: Biol Sex Differ. 2026 May 20;17:133. doi: 10.1186/s13293-026-00925-6 (PMC13366657; doi:10.1186/s13293-026-00925-6)
Supplement: Supplementary file 2 — Supplementary Material 2. [file 13293_2026_925_MOESM2_ESM.docx]

**Fig. S1 Physiological parameters of β-aminopropionitrile (BAPN)-treated mice by sex.** (**A**) Body weight changes of mice across different experimental groups over a 4-week period. (**B**) Weekly total water intake and (**C**) Body weight-corrected water intake in male and female BAPN-treated mice.


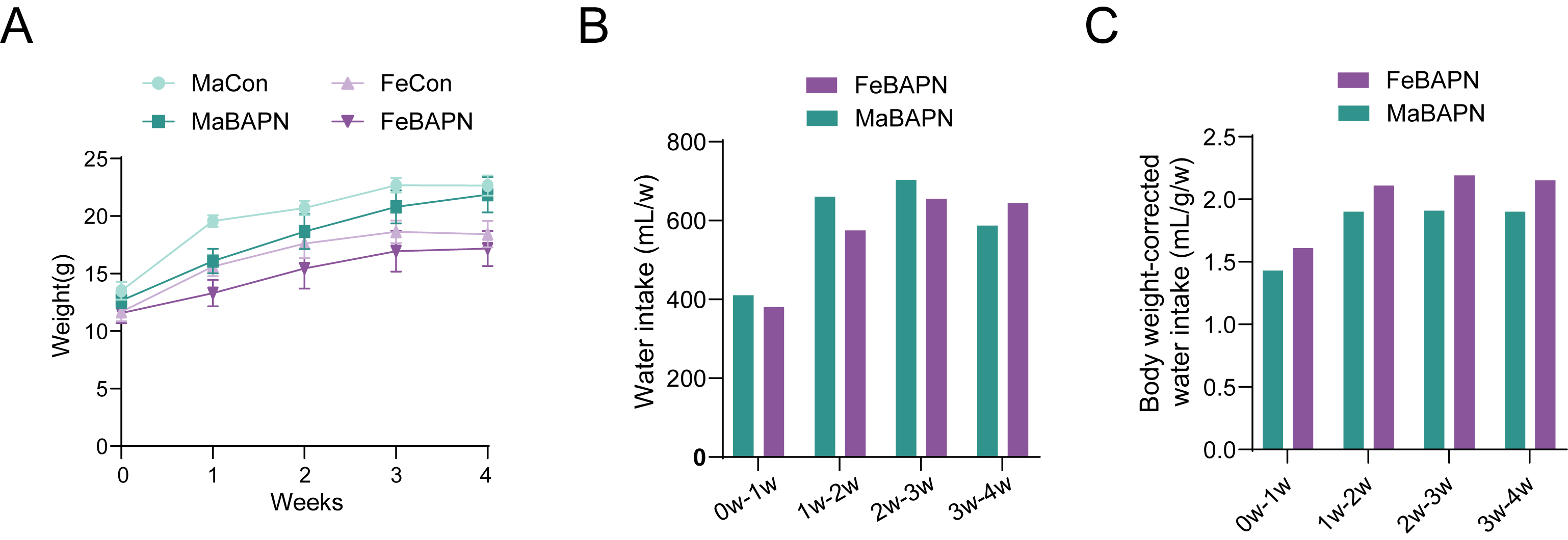


**Fig. S2 Microbial community structure and functional gene validation in β-aminopropionitrile (BAPN)-treated mice by sex.** Principal coordinate analysis (PCoA) plots based on (**A**) Weighted UniFrac distance and (**B**) Unweighted UniFrac distance, both calculated using OTU-level profiles; overall group differences were confirmed by PERMANOVA (adjusted *p* = 0.001), with corresponding pairwise PERMANOVA and PERMDISP results presented in Supplementary Tables 1 and 2. (**C**) PCoA plot based on Bray-Curtis distance calculated using amplicon sequence variants (ASVs). (**D**) Bar chart of linear discriminant analysis (LDA) scores from Linear Discriminant Analysis Effect Size (LEfSe) analysis identifying differentially abundant taxa across groups based on ASVs inferred using the DADA2 pipeline. (**E**) Log2-fold change (Log2FC) of key enzymes involved in the tryptophan–indole metabolic pathway across multiple comparison groups, based on functional predictions inferred by PICRUSt2 using ASV-level profiles; grey bars represent differences that are not statistically significant.

Supplementary Table 1. PERMANOVA and PERMDISP results of pairwise comparisons of fecal microbial profiles based on Weighted UniFrac distance across groups.

| comparison | R2 | *p*-value | adjusted  *p*-value (BH) | PERMDISP adjusted  *p* BH-p |
| --- | --- | --- | --- | --- |
| FeAAD vs FeCon | 0.15 | 0.011 | 0.028* | 0.779 |
| FeAAD vs FeNonAD | 0.04 | 0.696 | 0.696 | 0.156 |
| FeNonAD vs FeCon | 0.14 | 0.039 | 0.059 | 0.23 |
| MaAAD vs FeAAD | 0.16 | 0.021 | 0.038* | 0.033* |
| MaCon vs FeCon | 0.31 | 0.001 | 0.005** | 0.007** |
| MaCon vs MaAAD | 0.12 | 0.044 | 0.060 | 0.999 |
| MaCon vs MaNonAD | 0.19 | 0.023 | 0.038* | 0.143 |
| MaNonAD vs FeNonAD | 0.16 | 0.135 | 0.156 | 0.913 |
| MaNonAD vs MaAAD | 0.12 | 0.239 | 0.256 | 0.308 |

Note: PERMANOVA, Permutational Multivariate Analysis of Variance. The R² value represents the effect size (the proportion of variance explained by the group factor). PERMDISP, Permutation test for homogeneity of multivariate dispersions. Significance: * adjusted *p*-value < 0.05, ** adjusted *p*-value < 0.01. Abbreviations: Con, Control; AAD, Aortic Dissection or Aneurysm; NonAD, non- aortic dissection or aneurysm; Fe, Female; Ma, Male; BH, Benjamini–Hochberg correction.

Supplementary Table 2. PERMANOVA and PERMDISP results of pairwise comparisons of fecal microbial profiles based on Unweighted UniFrac distance across groups.

| comparison | R2 | *p*-value | adjusted  *p*-value(BH) | PERMDISP adjusted  *p* BH-p |
| --- | --- | --- | --- | --- |
| FeAAD vs FeCon | 0.15 | 0.011 | 0.028* | 0.015* |
| FeAAD vs FeNonAD | 0.04 | 0.696 | 0.696 | 0.119 |
| FeCon vs FeNonAD | 0.14 | 0.039 | 0.059 | 0.398 |
| MaAAD vs FeAAD | 0.16 | 0.021 | 0.038* | 0.003** |
| FeCon vs MaCon | 0.31 | 0.001 | 0.005** | 0.004** |
| FeNonAD vs MaNonAD | 0.16 | 0.135 | 0.156 | 0.015* |
| MaAAD vs MaCon | 0.12 | 0.044 | 0.060 | 0.134 |
| MaAAD vs MaNonAD | 0.12 | 0.239 | 0.256 | 0.003** |
| MaCon vs MaNonAD | 0.19 | 0.023 | 0.038* | 0.003** |

Note: PERMANOVA, Permutational Multivariate Analysis of Variance. The R² value represents the effect size (the proportion of variance explained by the group factor). PERMDISP, Permutation test for homogeneity of multivariate dispersions. Significance: * adjusted *p*-value < 0.05, ** adjusted *p*-value < 0.01. Abbreviations: Con, Control; AAD, Aortic Dissection or Aneurysm; NonAD, non- aortic dissection or aneurysm; Fe, Female; Ma, Male; BH, Benjamini–Hochberg correction.
